# Platform-Based Patient-Clinician Digital Health Interventions for Care Transitions: Scoping Review

**DOI:** 10.2196/55753

**Published:** 2024-12-30

**Authors:** Chantal Backman, Rosie Papp, Aurelie Tonjock Kolle, Steve Papp, Sarah Visintini, Ana Lúcia Schaefer Ferreira de Mello, Gabriela Marcellino de Melo Lanzoni, Anne Harley

**Affiliations:** 1 University of Ottawa Ottawa, ON Canada; 2 The Ottawa Hospital Ottawa, ON Canada; 3 Universidade Federal de Santa Catarina Florianópolis Brazil; 4 Bruyère Continuing Care Ottawa, ON Canada

**Keywords:** platform based, patient-clinician, digital health intervention, care transition, mobile phone

## Abstract

**Background:**

Care transitions are complex and can make patients vulnerable to adverse events. Poor communication among clinicians, patients, and their caregivers is a critical gap during these periods of transition. Technology solutions such as platform-based patient-clinician digital health interventions (DHIs) can provide support and education to patients.

**Objective:**

The aims of this scoping review were to explore the literature on platform-based patient-clinician DHIs specific to hospital-to-home care transitions and identify the barriers to and enablers of the uptake and implementation of these DHIs.

**Methods:**

A scoping review was conducted. A total of 4 databases (MEDLINE, CINAHL, Embase, and the Cochrane Central Register of Controlled Trials) were searched on July 13, 2022. Studies involving patients aged >18 years who used platform-based DHIs during their hospital-to-home transition were included. In total, 2 reviewers independently screened the articles for eligibility using a 2-stage process of title and abstract and full-text screening. Eligible studies underwent data extraction, and the results were analyzed using descriptive and narrative methods.

**Results:**

We screened 8322 articles, of which 97 (1.17%) met our inclusion criteria. DHIs were implemented using a mobile app (59/97, 61%), a web-based platform (28/97, 29%), or a combination of both (10/97, 10%). The 2 most common health conditions related to the DHIs were cardiac disease (22/97, 23%) and stroke (11/97, 11%). Outcomes varied greatly but were grouped by health care use, complications, and wellness outcomes. The top 2 barriers were lack of interest (13/97, 13%) and time constraints to use the DHIs (10/97, 10%), and the top 2 enablers were the ability to use the DHIs (17/97, 18%) and their ease of use (11/97, 11%). The main conflicting theme was access (enabler; 28/97, 29%) or limited access (barrier; 15/97, 15%) to technology or the internet.

**Conclusions:**

Platform-based DHIs could help improve communication, coordination, and information sharing between clinicians and patients during transition periods. Further research is needed to assess the effectiveness of these platform-based DHIs on patient outcomes.

## Introduction

Care transitions occur when a patient moves from one health care setting to another, such as from hospital to home. Effective care transitions are critical for ensuring that patients receive safe, high-quality care and for reducing the risk of adverse events, such as medication errors or unnecessary hospital readmissions [1]. However, care transitions are often complex and often involve multiple health care providers. In addition, recently hospitalized patients typically experience a temporary period of generalized risk of a wide range of adverse health events, also called “posthospital syndrome” [2]. Consequently, care transitions can be a significant source of errors, delays, and gaps in care [3,4]. One critical gap that has been identified is poor communication among clinicians, patients, and their caregivers [5,6], which is the one aspect that we hypothesize can be addressed through better technology solutions.

Technological solutions that can help improve the transition of patients between different care settings or health care providers are often referred to as “platform-based patient-clinician digital health interventions (DHIs).” These interventions can be used to address challenges during care transitions by leveraging technology to improve communication, coordination, and information sharing between clinicians and patients. These interventions can include but are not limited to providing patients with (1) personalized care plans, reminders, and educational resources to support self-management during transitions [7]; (2) remote monitoring to help health care providers track patient vital signs and symptoms to detect early warning signs of complications [8]; and (3) exchange and communication of information between clinicians and patients [9].

Previous research has identified 10 key elements in an ideal care transition [10]. The key domains of the Ideal Transition of Care framework [10] comprise (1) discharge planning; (2) complete communication of information; (3) availability, timeliness, clarity, and organization of information; (4) medication safety; (5) patient education to promote self-management; (6) social and community supports; (7) advanced care planning; (8) coordination of care among team members; (9) symptom monitoring and management after discharge; and (10) outpatient follow-ups. This framework was created to establish the necessary considerations pertaining to safe transitions in care. Ideally, any DHIs being developed would consider these proposed domains.

Platform-based patient-clinician DHIs can also facilitate continuity of care. Continuity of care is an important aspect that is present when a patient experiences coherent and linked care over time or when discrete elements of care that endure over time are maintained and supported [11]. Although continuity of care can be interpreted differently between care providers, there are 3 types that are agreed upon. The first type is “informational continuity,” which refers to the use of data from previous events in a patient’s medical history to inform the appropriate care of the patient’s current encounter [11]. The second type is “management continuity,” which occurs when care from multiple health care providers is linked in a coherent manner [11]. The third type is “relational continuity,” which acknowledges the importance of the relationship between patients and providers [11].

In addition, platform-based patient-clinician DHIs for care transitions have the potential to encourage individual behavior change and reduce health care costs [12]. It is important to ensure that these interventions are evidence based, user-friendly, and aligned with patient preferences and needs. There are limited reviews on DHIs for discharge and transitional care use, and these reviews focus mainly on a specific health aspect or condition, such as self-care associated with surgery [9] or postsurgery care for patients with hip fracture [13]. One other review identified specific health care provider roles and functions related to the use of DHIs [14]. Given that patients often have multiple health conditions, there are, to our knowledge, no reviews to date examining the use of DHIs during care transitions across various conditions to reduce siloed care. The aims of this scoping review were to explore the literature on platform-based patient-clinician DHIs specific to hospital-to-home care transitions and identify the barriers to and enablers of the uptake and implementation of these platform-based patient-clinician DHIs.

## Methods

### Design

We conducted a scoping review based on the work by Arksey and O’Malley [15], Levac et al [16], and the Joanna Briggs Institute scoping review methodologies [17]. The study is reported according to the PRISMA-ScR (Preferred Reporting Items for Systematic Reviews and Meta-Analyses extension for Scoping Reviews) checklist [18] (Multimedia Appendix 1). The methods involved five steps: (1) identifying the research question; (2) identifying relevant studies; (3) selecting the studies; (4) charting the data; and (5) collating, synthesizing, and reporting the results. The detailed protocol has been published previously [19].

### Search

A peer-reviewed search [20] was conducted on July 13, 2022, in MEDLINE, CINAHL, Embase, and the Cochrane Central Register of Controlled Trials (Multimedia Appendix 2). The main search concepts comprised terms related to “hospital to home transition,” “patient discharge,” “transitional care,” “internet-based interventions,” “mobile applications,” “mhealth,” and “digital health” platforms and were informed by previously conducted systematic searches [14,21,22]. No limit to language was applied; however, the results were limited by a publication date from 2012 onward. We chose to review results from the previous 10 years, recognizing the rapid pace of technological advancements. Search results were exported to Covidence (Veritas Health Innovation), a systematic review software [23], and duplicates were removed using the platform’s duplicate identification features.

### Screening

Studies were screened in 2 steps (title and abstract, and full text) based on the eligibility criteria (Textbox 1).

**Inclusion and exclusion criteria.**

**Inclusion criteria**

- Population: adult patients (aged >18 y) discharged from hospital to home
- Concept: digital-based platforms that support a hospital-to-home transition, including web-based digital health interventions (DHIs), defined as programs that were delivered via the internet and accessed through a website link (URL) [24], and mobile apps, defined as software programs developed for smartphones [25]
- Context: hospital-to-home transitions
- Type of evidence: studies published after 2012 and identified as randomized controlled trials, quasi-experimental studies, pilot studies, feasibility studies, observational studies (case-control, cohort, cross-sectional, and descriptive studies), or qualitative studies

**Exclusion criteria**

- Concept: non–platform-based DHIs, including but not limited to wearable devices if the intervention was stand-alone (eg, to track activity), prosthetics, robotics, medical imaging technology (eg, x-rays and ultrasounds), interventions using only a standard telephone, machine learning, and telehealth
- Type of evidence: studies in the design stage at the time of screening or if the platform-based patient-clinician DHIs were tailored specifically to and solely focused on mental health or cancer as these are unique clinical areas

### Data Charting

Studies that met all the inclusion criteria were extracted using the Google Forms platform. The pilot-tested Google Form used a survey format for the extraction of the relevant information, including the following: lead author, year of publication, country, objectives, study design, participants, patient health condition, name of DHI, rationale of intervention, theory guiding the intervention, content of the intervention, elements of postcare, digital health tools, function of digital health tool, management or informational or relational continuity of care [11], who provided the intervention, number of days after discharge, number and duration of sessions, tailoring or modification, adherence or attrition, results, and barriers or enablers. To ensure a high-quality description of the interventions, the information extracted from each study was guided by the recommendations made by the 12-item Template for Intervention Description and Replication [26]. After data charting was completed, the Google Form was used to generate a Microsoft Excel spreadsheet to analyze the findings using descriptive and narrative methods.

## Results

### Overview

**Figure 1:**
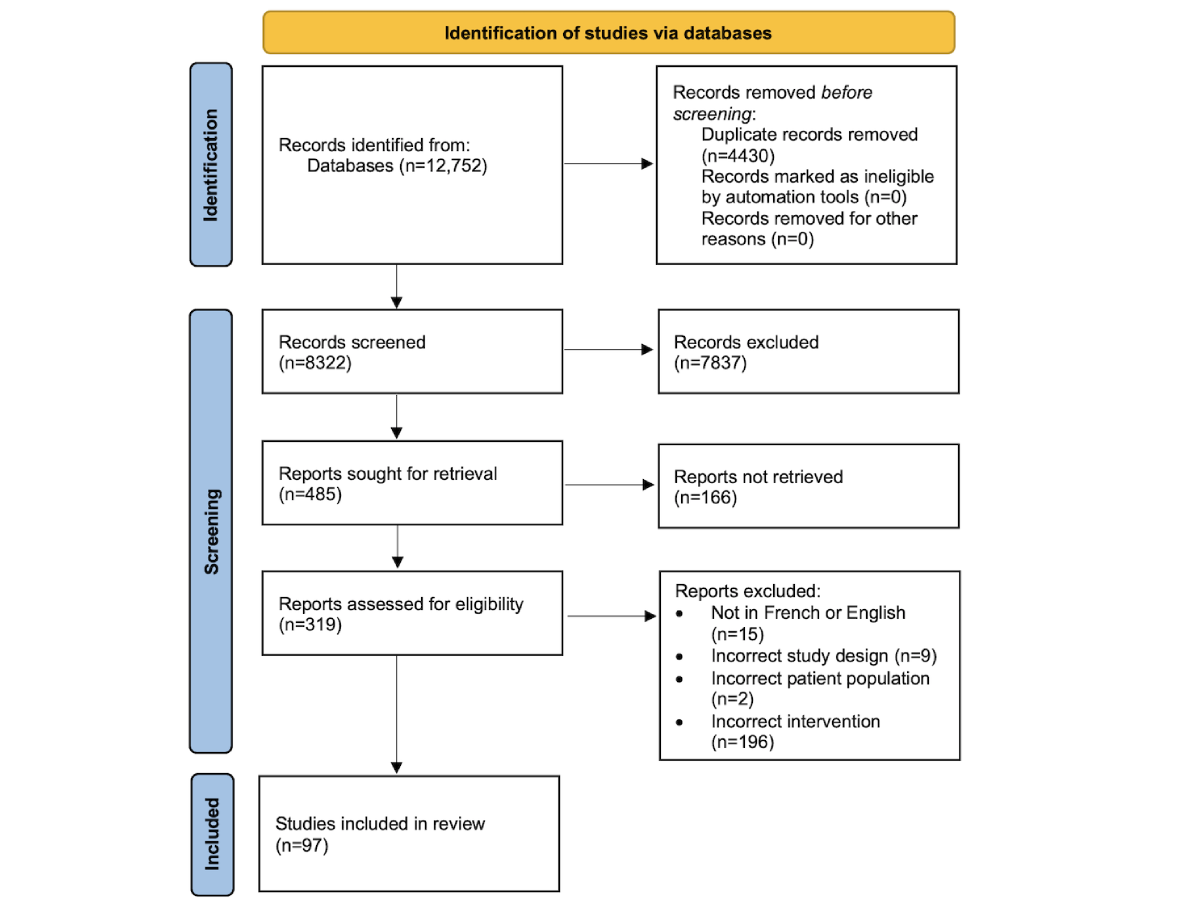
PRISMA (Preferred Reporting Items for Systematic Reviews and Meta-Analyses) flow diagram—digital heath interventions (DHIs) for care transitions.

A total of 12,752 records were retrieved from the search, of which 4430 (34.74%) were duplicates and 7837 (61.46%) were rejected at the abstract review stage, leaving 485 (3.8%) records selected for full-text review. A total of 34.2% (166/485) of the reports could not be retrieved as the full text was unavailable. In addition, 222 full-text articles were excluded for the following reasons: incorrect intervention (not DHIs related to care transitions; n=196, 88.3%), language other than French or English (n=15, 6.8%), incorrect study design (n=9, 4.1%), and incorrect patient population (n=2, 0.9%). The list of articles with reasons for exclusion can be found in Multimedia Appendix 3. The final review included a total of 97 studies after the assessment process was completed (Figure 1 [27]).

### Characteristics of the Included Studies

Of the 97 included studies [28-124], 34 (35%) were conducted in the United States [31,38,43,48,49,53,54,56,59,61-63, 67,71,73-75,78,79,87,89,90,92,93,102,104-108,111,113,116,118], 16 (16%) were conducted in Canada [30,33,36,51,52,55,57,60,70,80,81,94,96,97,100,117], 10 (10%) were conducted in China [41,50,82-85,91,109,121,122], 8 (8%) were conducted in the Netherlands [39,40,76,95,103,112,119,120], and 5 (5%) were conducted in Australia [42,66,68,101,115]. The remaining 25% (24/97) of the studies were conducted in 14 other countries [28,29,32,34,35,37,44-47,58,64,65,69,72,77,86,88,98,99,110,114,123,124]. The 2 most common health conditions relevant to the platform-based patient-clinician DHIs were cardiac disease (22/97, 23%) [29,31,36,38,49,50,58,59,65-67,72,79,83,87, 89,90,97,109,114,116,123] and stroke (11/97, 11%) [32,44,71,96,98,104,107,110,115,119,124].

A wide variety of elements of postcare were implemented in the platform-based patient-clinician DHIs, including but not limited to detecting postoperative issues, assessing patients’ needs, improving patient understanding, rehabilitation, improving symptom management, and increasing self-care (Table 1).

**Table 1:** Study characteristics.

Study	Country	Study design	Setting	Participants	Health condition	Name of DHI^a^	Digital health tool	Elements of postcare
Agri et al [28], 2020	Switzerland	Retrospective monocentric cohort study	Community or home or retirement home	Patients—intervention: n=43	Colorectal surgery (colorectal resections, ostomy procedures, and stoma closures)	Maela	Mobile app	Enhancing patient-provider communication; ability to identify symptoms of poor wound healing; enhancing patients’ knowledge, skills, and confidence; detecting any postoperative issues; pain control or management; wound care
Antypas and Wangberg [29], 2014	Norway	Randomized controlled trial	Community, home, or retirement home	Patients—intervention: n=7; control: n=12	Cardiovascular disease	Internet- and mobile-based tailored intervention to enhance maintenance of physical activity after cardiac rehabilitation	Mobile app and web app	Improving physical activity
Armstrong et al [30], 2017	Canada	Randomized controlled trial	Community, home, or retirement home	Patients—intervention: n=32; control: n=33	Ambulatory breast reconstruction surgery	QoC^b^ Health Inc mobile app	Mobile app	Ability to identify symptoms of poor wound healing; detecting any postoperative issues; improving symptom management; follow-up appointments with primary care provider; pain control or management; wound care
Athilingam et al [31], 2017	United States	Feasibility (pilot) study; randomized controlled trial	Community, home, or retirement home	Patients—intervention: n=9; control: n=9	Congestive HF^c^	Mobile app to improve self-care behaviors and quality of life for patients with HF	Mobile app	Increasing self-care; medication management; fostering treatment adherence; improving quality of life; enhancing HF-specific knowledge
Avci and Gozum [32], 2018	Turkey	Descriptive study	Community, home, or retirement home	Caregivers—intervention: n=62	Stroke	Supportive website for the caregivers of patients with stroke after discharge	Web app	Enhancing caregiver preparedness; enhancing caregiver knowledge and skills
Backman et al [33], 2020	Canada	Feasibility (pilot) study	Community, home, or retirement home	Patients: n=34; caregivers: n=19; clinicians: n=37	Hip fracture	MyPath to Home	Web app	Promoting communication among patients, caregivers, and clinicians
Bäcker et al [34], 2021	Germany	Randomized controlled trial	Community, home, or retirement home	Patients—intervention: n=20; control: n=15	Knee arthroplasty	GenuSport	Mobile app	Rehabilitation (physical therapy and occupational therapy)
Bauwens et al [35], 2022	France	Case-control	Community, home, or retirement home	Patients—intervention: n=32; control: n=101	ACL^d^ reconstruction surgery	Doct-Up	Mobile app	Encouraging ambulation; pain control or management; rehabilitation (physical therapy and occupational therapy)
Ben-Ali et al [36], 2021	Canada	Feasibility (pilot) study	Community, home, or retirement home	Patients—intervention: n=1108	Cardiac surgery	SeamlessMD	Mobile app	Enhancing patients’ knowledge, skills, and confidence; improving patient understanding; increasing self-care; detecting any postoperative issues; encouraging lifestyle changes; improving symptom management; pain control or management; wound care; providing PROs^e^, surveys, and feedback
Birkhäuser et al [37], 2020	Switzerland	Prospective nonrandomized pilot clinical trial	Community, home, or retirement home	Patients—intervention: n=18	Radical cystectomy	Cellphone-based health care app	Mobile app and web app	Monitor progression of patient recovery
Blewer et al [38], 2020	United States	Randomized controlled trial	Community, home, or retirement home	Patients—intervention: n=699; control: n=626	Coronary artery disease	mApp CPR^f^ training app	Mobile app	Providing effective CPR
Bouwsma et al [39], 2018	The Netherlands	Randomized controlled trial	Community, home, or retirement home	Patients—intervention: n=227; control: n=206	Hysterectomy and laparoscopic adnexal surgery	eHealth intervention	Web app	Ability to identify symptoms of poor wound healing; enhancing patients’ knowledge, skills, and confidence; detecting any postoperative issues; improving symptom management; guidance in the process of resuming work activities
Bouwsma et al [40], 2018	The Netherlands	Randomized controlled trial	Community, home, or retirement home	Patients—intervention: n=227; control: n=206	Hysterectomy or laparoscopic adnexal surgery	eHealth intervention	Web app	Enhancing patients’ knowledge, skills, and confidence; detecting any postoperative issues; encouraging return to work
Cheng et al [41], 2022	China	Randomized controlled trial	Community, home, or retirement home	Patients—intervention: n=19; control: n=20	Hip fracture	Home-based rehabilitation mobile app	Mobile app	Enhancing caregiver knowledge and skills; enhancing patients’ knowledge, skills, and confidence; improving physical activity; rehabilitation (physical therapy and occupational therapy); fostering treatment adherence; progress summary (can track completion of tasks), reminders, and support information
Cox et al [42], 2015	Australia	Feasibility (pilot) study	Community, home, or retirement home	Patients—intervention: n=10	Cystic fibrosis	ActivOnline	Web app	Improving physical activity; encouraging lifestyle changes; improving symptom management; improving quality of life
Davis et al [43], 2020	United States	Retrospective review	Community, home, or retirement home	Patients—intervention: n=47	Total shoulder arthroplasty	Force Therapeutics	Mobile app	Enhancing patient-provider communication; enhancing patients’ knowledge, skills, and confidence; rehabilitation (physical therapy and occupational therapy)
Davoody and Hägglund [44], 2016	Sweden	Qualitative study	Community, home, or retirement home	Care professionals—intervention: n=8	Stroke	eHealth for postdischarge stroke	Web app	Improve patient understanding; rehabilitation (physical therapy and occupational therapy)
De Batlle et al [45], 2021	Spain	Prospective, pragmatic, 2-arm, parallel-implementation trial	Community, home, or retirement home	Patients—intervention: n=48; control: n=28	Chronic obstructive pulmonary disease and HF	CONNECARE	Mobile app and wearable device	Enhancing patient-provider communication; monitoring pulse, oxygen, HR^g^, BP^h^, and weight at home; enhancing patients’ knowledge, skills, and confidence; improving physical activity; assessing patients’ needs
Debono et al [46], 2016	France	Feasibility (pilot) study	Community, home, or retirement home	Patients—intervention: n=60	Ambulatory lumbar discectomy	Mobile app for postoperative monitoring after outpatient lumbar discectomy	Mobile app	Enhancing patient-provider communication; assessing rates of complications; pain control or management
Debono et al [47], 2019	France	Retrospective analysis	Community, home, or retirement home	Patients—intervention: n=1920; control: n=1563	Spinal cord injury and lumbar disc herniation	e-fitback	Mobile app	Enhancing patient-provider communication; detecting any postoperative issues; pain control or management; wound care
Devito Dabbs et al [48], 2016	United States	Randomized controlled trial	Community, home, or retirement home	Patients—intervention: n=99; control: n=102	Lung transplant	Pocket PATH^i^	Mobile app	Detecting any postoperative issues; assessing patients’ needs; assessing rates of complications; pain control or management
Dorsch et al [49], 2021	United States	Randomized controlled trial	Community, home, or retirement home	Patients—intervention: n=42; control: n=41	HF	ManageHF4Life	Mobile app and monitoring devices	Enhancing patients’ knowledge, skills, and confidence; increasing self-care; encouraging lifestyle changes; improving symptom management; assessing patients’ needs; medication management; nutrition support; improving quality of life
Duan et al [50], 2018	China	Pilot randomized controlled trial	Community, home, or retirement home	Patients—intervention: n=44; control: n=39	Coronary artery disease	Health behavior intervention for patients with coronary heart disease through the web	Web app	Improving physical activity; improving food consumption
Dukeshire et al [51], 2012	Canada	Feasibility (pilot) study	Community, home, or retirement home	Patients—intervention: n=31	Hysterectomy	SAFER^j^ project	Web app	Improving symptom management; ability to identify symptoms; postsurgical care
Eustache et al [52], 2023	Canada	Cohort study	Community, home, or retirement home	Patients—intervention: n=94; matched cohort: n=256	Colorectal surgery	Same-day discharge mHealth^k^ app (CareSense)	Mobile app	Enhancing patients’ knowledge, skills, and confidence; detecting any postoperative issues; enhancing patient-provider communication
Felbaum et al [53], 2018	United States	Prospective cohort study	Community, home, or retirement home	Patients—intervention: n=56	Spinal cord injury, lumbar disc herniation, and neurosurgery (spinal and cranial surgery)	TrackMyRecovery	Mobile app	Enhancing patient-provider communication; ability to identify symptoms of poor wound healing; detecting any postoperative issues; pain control or management; wound care
Ganapathy et al [54], 2017	United States	Feasibility (pilot) study	Community, home, or retirement home	Patients—intervention: n=40; caregivers—intervention: n=40	Cirrhosis and hepatic encephalopathy	Patient Buddy	Mobile app	Enhancing caregiver preparedness; enhancing caregiver knowledge and skills; enhancing patient-provider communication; monitoring pulse, oxygen, HR, BP, and weight at home; enhancing patients’ knowledge, skills, and confidence; detecting any postoperative issues; delirium screening and management; fall prevention; medication management; entering grams of sodium consumed; assessing cognition (EncephalApp); Timed Up and Go test
Gollish et al [55], 2019	Canada	Feasibility (pilot) study; qualitative study	Community, home, or retirement home	Patients—intervention: n=629	Total hip replacement and knee arthroplasty	myHip&Knee	Mobile app	Enhancing patient-provider communication; increasing self-care; detecting any postoperative issues; encouraging lifestyle changes; improving symptom management; medication management; pain control or management
Gunter et al [56], 2018	United States	Feasibility (pilot) study	Community, home, or retirement home	Patients—intervention: n=40	Vascular surgery	WoundCheck	Mobile app	Ability to identify symptoms of poor wound healing; detecting any postoperative issues; improving symptom management; wound care
Habib et al [57], 2021	Canada	Randomized controlled trial	Community, home, or retirement home	Patients—intervention: n=23; control: n=26	All health conditions	SAM^l^	Mobile app	Medication management; promoting adherence to medication
Hägglund et al [58], 2015	Sweden	Randomized controlled trial	Community, home, or retirement home	Patients—intervention: n=32; control: n=40	HF	Home intervention system (OPTILOGG^m^)	Web app and monitoring devices	Enhancing patient-provider communication; enhancing patients’ knowledge, skills, and confidence; improving patient understanding; increasing self-care; improving symptom management
Heiney et al [59], 2020	United States	Feasibility (pilot) study	Community, home, or retirement home	Patients—intervention: n=12	HF	Healthy Heart	Mobile app	Enhancing patient-provider communication; monitoring pulse, oxygen, HR, BP, and weight at home; ability to identify symptoms of HF; enhancing patients’ knowledge, skills, and confidence; encouraging lifestyle changes; medication management; managing emotional changes; improving quality of life
Heuser et al [60], 2021	Canada	Retrospective cohort study	Community, home, or retirement home	Patients—intervention: n=396; control: n=458	Obesity and bariatric surgery	SeamlessMD	Mobile app	Managing mood and anxiety; enhancing patients’ knowledge, skills, and confidence; increasing self-care; improving physical activity; encouraging lifestyle changes; improving symptom management; medication management; nutrition support; wound care
Heyworth et al [61], 2014	United States	Feasibility (pilot) study	Community, home, or retirement home	Patients—intervention: n=60	All health conditions	SMMRT^n^	Web app	Medication management
Highland et al [62], 2019	United States	Feasibility (pilot) study; randomized controlled trial	Community, home, or retirement home	Patients—intervention: n=24; control: n=26	Peripheral nerve block affecting one or more of the limbs	mCare system	Mobile app	Detecting any postoperative issues; assessing patients’ needs
Holzer et al [63], 2022	United States	Feasibility (pilot) study	Community, home, or retirement home, rehabilitation, long-term care and nursing home (24-hour care)	Patients—intervention: n=89; control: n=128	Acute venous thromboembolism	HealthFlo	Mobile app	Enhancing patient-provider communication; enhancing patients’ knowledge, skills, and confidence; medication management
Houchen-Wolloff et al [64], 2021	United Kingdom	Feasibility (pilot) study	Community, home, or retirement home	Patients—intervention: n=100	Chronic obstructive pulmonary disease	SPACE^o^ for chronic obstructive pulmonary disease	Web app	Enhancing patient-provider communication; improving patient understanding; increasing self-care; improving physical activity
İlaslan and Özer [65], 2022	Turkey	Randomized controlled trial	Community, home, or retirement home	Patients—intervention: n=32; control: n=32	Congestive HF	Web app for training and telephone follow-up for patients with HF	Web app	Ability to identify symptoms of HF; improving symptom management; meeting the informational needs of patients
Indraratna et al [66], 2022	Australia	Feasibility (pilot) study; randomized controlled trial	Community, home, or retirement home	Patients—intervention: n=81; control: n=83	Cardiac disease	TeleClinical Care	Mobile app and wearable device	Monitoring pulse, oxygen, HR, BP, and weight at home
Johnson et al [67], 2022	United States	Feasibility (pilot) study	Community, home, or retirement home	Patients—intervention: n=16; control: n=15	Decompensated HF	HF-SMART^p^	Web app	Ability to identify symptoms of HF; enhancing patients’ knowledge, skills, and confidence; improving patient understanding; encouraging lifestyle changes; improving symptom management; improving quality of life
Kang et al [68], 2022	Australia	Feasibility (pilot) study; randomized controlled trial	Community, home, or retirement home	Patients—intervention: n=43; control: n=42	General surgery	Web-based discharge education program	Web app	Increasing self-care
Kargar et al [69], 2020	Iran	Randomized controlled trial	Community, home, or retirement home	Patients—intervention: n=30; control: n=30	Burns	Self-care educational mobile app for burns	Mobile app and web app	Improving quality of life
Keng et al [70], 2020	Canada	Cross-sectional study	Community, home, or retirement home	Patients—intervention: n=106	Colorectal surgery	Home to Stay digital program after colorectal surgery	Mobile app	Monitoring patient recovery at home
Kersey et al [71], 2022	United States	Feasibility (pilot) study; randomized controlled trial	Community, home, or retirement home	Patients—intervention: n=16; control: n=15	Stroke	mHealth platform for strategy training in inpatient stroke rehabilitation (iADAPT^q^)	Mobile app	Assessing the feasibility of inpatient stroke rehabilitation
Khan et al [72], 2018	Denmark	Mixed methods study	Community, home, or retirement home	Patients—intervention: n=33	Cardiac surgery	Activeheart portal	Web app	Not reported
Khanwalkar et al [73], 2019	United States	Case-control	Community, home, or retirement home	Patients—intervention: n=208	Septosplasty and endoscopic sinus surgery	DPE^r^ platform	Mobile app	Detecting any postoperative issues; pain control or management; PROs; monitoring pain; collecting data on the postoperative day when the patient returned to work
Kim et al [74], 2016	United States	Feasibility (pilot) study	Community, home, or retirement home	Patients—intervention: n=13	Knee arthroplasty	iGB^s^ program	Mobile app	Enhancing patients’ knowledge, skills, and confidence; increasing self-care; detecting any postoperative issues; improving physical activity; encouraging lifestyle changes; rehabilitation (physical therapy and occupational therapy); improving quality of life
Knapp et al [75], 2021	United States	Quantified patient engagement	Community, home, or retirement home	Patients—intervention: n=207	Total hip replacement and knee arthroplasty	PeerWell	Mobile app, web app, and SMS text messaging	Promoting mental well-being; enhancing patients’ knowledge, skills, and confidence; improving physical activity; nutrition support; rehabilitation (physical therapy and occupational therapy); improving quality of life
Kooij et al [76], 2021	The Netherlands	Feasibility (pilot) study; mixed methods study	Community, home, or retirement home	Patients: n=39	Chronic obstructive pulmonary disease	Self-management app for high-risk patients with chronic obstructive pulmonary disease	Mobile app	Increasing self-care; improving symptom management; medication management
Kristjánsdóttir et al [77], 2013	Norway	Randomized controlled trial	Community, home, or retirement home	Patients—intervention: n=48; control: n=64	Chronic widespread pain	Smartphone-based intervention for chronic widespread pain	Mobile app and web app	Promoting self-management of chronic pain
Kummerow et al [78], 2015	United States	Feasibility (pilot) study	Community, home, or retirement home	Patients—intervention: n=50	General surgery	MHAV^t^	Web app	Enhancing patient-provider communication; improving symptom management; assessing patients’ needs; wound care
Layton et al [79], 2014	United States	Feasibility (pilot) study	Community, home, or retirement home	Patients—intervention: n=16	Coronary artery disease and congestive HF	Smartphone-based app to monitor outpatient discharge instructions of patients with cardiac disease	Mobile app	Enhancing patient-provider communication; improving patient understanding; increasing self-care; follow-up appointments with primary care provider; medication management; encouraging activity
Lee et al [80], 2022	Canada	Feasibility (pilot) study	Community, home, or retirement home	Patients—intervention: n=70; control: n=35	Colorectal surgery	Mobile app follow-up for same-day discharge	Mobile app	Detecting any postoperative issues; pain control or management
Lee et al [81], 2022	Canada	Cohort study	Community, home, or retirement home	Patients—intervention: n=48; control: n=73	Colorectal surgery	mHealth remote postdischarge monitoring	Mobile app	Enhancing patient-provider communication; improving symptom management; assessing rates of complications
Liu et al [82], 2021	China	Randomized controlled trial	Community, home, or retirement home	Patients—intervention: n=49; control: n=49	Spinal cord injury	Together	Mobile app	Enhancing patients’ knowledge, skills, and confidence; detecting any postoperative issues; follow-up appointments with primary care provider; improving quality of life
Li et al [83], 2022	China	Randomized controlled trial	Community, home, or retirement home	Patients—intervention: n=143; control: n=147	Coronary artery disease	DTx^u^	Mobile app	Monitoring pulse, oxygen, HR, BP, and weight at home; enhancing patients’ knowledge, skills, and confidence; encouraging lifestyle changes; improving symptom management; medication management
Lou et al [84], 2022	China	Quasi-experimental study	Community, home, or retirement home	Patients—intervention: n=101; control: n=60	Not reported	mVS^v^ intervention to enhance spiritual well-being	Mobile app	Fostering spiritual well-being
Lyu et al [85], 2021	China	Randomized controlled trial	Community, home, or retirement home	Patients—intervention: n=58; control: n=58	Diabetes (type 2)	Nurse-led web-based transitional care program	Web app	Increasing self-care; fostering treatment adherence; improving quality of life
María Gómez et al [86], 2022	Colombia	Randomized controlled trial	Community, home, or retirement home	Patients—intervention: n=39; control: n=42	Diabetes (type 2)	mHealth app for patients with type 2 diabetes	Mobile app and web app	Managing glycemic control
Marvel et al [87], 2021	United States	Nonrandomized controlled trial	Community, home, or retirement home	Patients—intervention: n=200; control: n=864	Acute myocardial infarction	Acute myocardial infarction DHI	Mobile app and wearable device	Enhancing patient-provider communication; monitoring pulse, oxygen, HR, BP, and weight at home; increasing self-care; follow-up appointments with primary care provider; medication management
Metilda et al [88], 2021	India	Randomized controlled trial	Community, home, or retirement home	Patients—intervention: n=50; control: n=50	Brain injury	Aimeo	Mobile app	Enhancing patient-provider communication; enhancing patients’ knowledge, skills, and confidence; detect any postoperative issues
Park et al [89], 2019	United States	Feasibility and adoptability study	Community, home, or retirement home	Patients—intervention: n=58	Congestive HF	Digital health monitoring for patients with HF	Mobile app and web app	Ability to identify symptoms of HF; increasing self-care; improving symptom management
Paruchuri et al [90], 2021	United States	Feasibility (pilot) study	Community, home, or retirement home	Patients—intervention: n=118; control: n=343	Coronary artery disease	Wellframe	Mobile app	Enhancing patients’ knowledge, skills, and confidence; encouraging lifestyle changes; improving symptom management; improving quality of life
Peng et al [91], 2022	China	Randomized controlled trial	Community, home, or retirement home	Patients—intervention: n=47; control: n=47	Hepato-pancreato-biliary surgery and biliary tract disease	Mobile continuous nursing platform	Mobile app	Enhancing caregiver knowledge and skills; enhancing patient-provider communication; ability to identify symptoms of poor wound healing; enhancing patients’ knowledge, skills, and confidence; improving caregiver understanding; detecting any postoperative issues; assessing patients’ needs; medication management; managing emotional changes; nutrition support; improving quality of life; wound care; T-tube placement and fixation method; observation of bile-related traits; treatment method of T-tube slippage; selection, fixation, or replacement of drainage bag
Pickens et al [92], 2019	United States	Feasibility (pilot) study	Community, home, or retirement home	Patients—intervention: n=122	Hepato-pancreato-biliary surgery	SeamlessMD	Mobile app	Managing mood and anxiety; enhancing patients’ knowledge, skills, and confidence; detecting any postoperative issues; improving physical activity; improving symptom management; nutrition support; improving quality of life; collecting PROs
Ponder et al [93], 2020	United States	Descriptive study; feasibility (pilot) study	Community, home, or retirement home	Patients—intervention: n=47	Spinal cord injury	Smartphone app with a digital care pathway for patients undergoing spine surgery	Mobile app and web app	Improving patient engagement; facilitating shared decision-making between patients and caregivers
Pooni et al [94], 2022	Canada	Randomized controlled trial	Community, home, or retirement home	Patients—intervention: n=128; control: n=125	Colorectal surgery	Postdischarge Home to Stay mobile app	Mobile app and web app	Ability to identify symptoms of poor wound healing; improving patient understanding; detecting any postoperative issues
Pronk et al [95], 2020	The Netherlands	Randomized controlled trial	Community, home, or retirement home	Patients—intervention: n=38; control: n=33	Knee arthroplasty	PainCoach app	Mobile app	Medication management; pain control or management; rehabilitation (physical therapy and occupational therapy)
Pugliese et al [96], 2019	Canada	Feasibility (pilot) study	Community, home, or retirement home	Patients—intervention: n=30	Stroke	RecoverNow	Mobile app	Enhancing patient-provider communication; rehabilitation (physical therapy and occupational therapy)
Reid et al [97], 2012	Canada	Randomized controlled trial	Community, home, or retirement home	Patients—intervention: n=115; control: n=118	Cardiac disease	CardioFit internet-based expert system	Web app	Improving physical activity
Requena et al [98], 2019	Spain	2-arm open-label nonrandomized study	Community, home, or retirement home	Patients—intervention: n=107; control: n=52	Stroke	FARMALARM	Mobile app	Enhancing patient-provider communication; enhancing patients’ knowledge, skills, and confidence; improving physical activity; medication management; controlling vascular risk factors
Rian et al [99], 2022	Norway	Randomized controlled trial	Community, home, or retirement home	Patients—intervention: n=134	Knee arthroplasty	Eir (Eir Solutions AS)	Web app	Enhancing patient-provider communication; detecting any postoperative issues; medication management
Rosner et al [100], 2018	Canada	Cohort study	Community, home, or retirement home	Patients—intervention: n=371	Orthopedic fracture (any)	Internet-based orthopedic patient self-reports of postdischarge complications	Mobile app	Enhancing patient-provider communication; improving symptom management; assessing rates of complications
Saunders et al [101], 2021	Australia	Randomized controlled trial	Community, home, or retirement home	Patients—intervention: n=50; control: n=49	Hip osteoarthritis	My Hip Journey	Web app and email reminders	Enhancing caregiver preparedness; enhancing patient-provider communication; enhancing patients’ knowledge, skills, and confidence; improving patient understanding; increasing self-care; improving physical activity; encouraging lifestyle changes; improving quality of life
Schenkel et al [102], 2020	United States	Feasibility (pilot) study	Community, home, or retirement home	Patients—intervention: n=28; control: n=28	Lung transplantation	ActiCare	Web app	Enhancing caregiver knowledge and skills; enhancing patient-provider communication; monitoring pulse, oxygen, HR, BP, and weight at home; enhancing patients’ knowledge, skills, and confidence; increasing self-care; detecting any postoperative issues; encouraging lifestyle changes; medication management; nutrition support; rehabilitation (physical therapy and occupational therapy); tracking appointments
Scheper et al [103], 2019	The Netherlands	Cohort study	Community, home, or retirement home	Patients—intervention: n=69	Total hip replacement and knee arthroplasty	Woundcare	Mobile app	Enhancing patient-provider communication; ability to identify symptoms of poor wound healing; detecting any postoperative issues; pain control or management; wound care; prevention of prosthetic joint infection
Schneider and Howard [104], 2017	United States	Descriptive study	Community, home, or retirement home	Patients—intervention: n=44; control: n=42	Stroke	Technology-improved coping for patients after stroke	Mobile app	Improving patient understanding; improving symptom management; follow-up appointments with primary care provider; medication management; managing emotional changes
Schubart [105], 2012	United States	Feasibility (pilot) study	Community, home, or retirement home	Patients—intervention: n=14	Spinal cord injury	e-Learning program to prevent pressure ulcers in adults with spinal cord injury	Web app	Pressure ulcer prevention; pressure ulcer management
Scott et al [106], 2017	United States	Mixed methods study	Community, home, or retirement home	Patients—intervention: n=20	Colorectal surgery	Postoperative mHealth app	Mobile app	Not reported
Siegel et al [107], 2016	United States	Feasibility (pilot) study	Not reported	Patients—intervention: n=3	Stroke	PHA^w^ stroke app	Mobile app	Enhancing patient-provider communication; follow-up appointments with primary care provider; medication management
Stapler et al [108], 2022	United States	Case-control, retrospective analysis	Community, home, or retirement home	Patients—preintervention group: n=1052; postintervention group: n=668	Elective colon and rectal surgery; colorectal neoplasia, diverticulitis, IBD^x^, and other diseases of the colon and rectum	St. Joseph's Health App	Mobile app	Detecting any postoperative issues; nutrition support; rehabilitation (physical therapy and occupational therapy); wound care
Su and Yu [109], 2021	China	Randomized controlled trial	Community, home, or retirement home	Patients—intervention: n=73; control: n=73	Coronary heart disease	NeCR^y^	Web app	Promoting mental well-being; enhancing patients’ knowledge, skills, and confidence; increasing self-care; detecting any postoperative issues; improving physical activity; encouraging lifestyle changes; assessing patients’ needs; improving quality of life; cardiac rehabilitation
Sureshkumar et al [110], 2016	India	Feasibility (pilot) study	Community, home, or retirement home	Patients—intervention: n=60	Stroke	Care for Stroke intervention	Mobile app and web app	Enhancing functional skills and activities of daily living
Symer et al [111], 2017	United States	Feasibility (pilot) study	Community, home, or retirement home	Patients—intervention: n=31	Major abdominal surgery	Gastrointestinal mHealth app	Mobile app and wearable device	Managing mood and anxiety; ability to identify symptoms of poor wound healing; detecting any postoperative issues; improving symptom management; wound care; decreasing length of hospital stay
Timmers et al [112], 2019	The Netherlands	Randomized controlled trial	Community, home, or retirement home	Patients—intervention: n=114; control: n=99	Knee arthroplasty	The Patient Journey App	Mobile app	Enhancing patients’ knowledge, skills, and confidence; increasing self-care; pain control or management; rehabilitation (physical therapy and occupational therapy); improving quality of life; wound care
Tolentino [113], 2020	United States	Case-control, retrospective study	Community, home, or retirement home	Patients—intervention: n=50; control: n=50	Multiple conditions	Meducation	Mobile app	Medication management
Torri et al [114], 2018	Italy	Quasi-experimental study	Community, home, or retirement home	Patients—intervention: n=26; control: n=27	Coronary artery disease, cardiac surgery, congestive HF, percutaneous coronary revascularization, or acute ischemic events	CRMP^z^	Mobile app	Improving physical activity; encouraging lifestyle changes; medication management; improving quality of life
Van den Berg et al [115], 2016	Australia	Proof-of-concept trial	Community, home, or retirement home	Patients—intervention: n=31; control: n=32	Stroke	CARE4STROKE	Mobile app and wearable device	Enhancing patient-provider communication; improving physical activity; early weight bearing (weight bearing as tolerated); encouraging ambulation; rehabilitation (physical therapy and occupational therapy); improving quality of life
Venkatraman et al [116], 2022	United States	Feasibility (pilot) study	Community, home, or retirement home	Patients—intervention: n=69	Cardiac surgery and aortic stenosis	MMS^aa^	Mobile app	Ability to identify symptoms of HF; enhancing patients’ knowledge, skills, and confidence; increasing self-care; recording baseline and postoperative PROs
Vincent et al [117], 2021	Canada	Qualitative study; qualitative usability study	Community, home, or retirement home	Patients and caregivers—intervention: n=17	Hip fracture	My-HF^ab^	Mobile app	Enhancing patient-provider communication; managing mood and anxiety; enhancing patients’ knowledge, skills, and confidence
Visperas et al [118], 2021	United States	Randomized controlled trial	Community, home, or retirement home	Patients—intervention: n=204; control: n=195	Total hip replacement and knee arthroplasty	JointCOACH	Web app	Enhancing patient-provider communication; enhancing patients’ knowledge, skills, and confidence; detecting any postoperative issues; medication management; pain control or management; rehabilitation (physical therapy and occupational therapy)
Vloothuis et al [119], 2019	The Netherlands	Randomized controlled trial	Community, home, or retirement home	Intervention: n=32; control: n=34—patient-caregiver dyads	Stroke	CARE4STROKE digital intervention	Web app	Managing mood and anxiety; increasing self-care; improving physical activity; improving quality of life; improving motor impairment, strength, walking ability, balance, mobility, and (extended) activities of daily living of patients; reducing caregiver strain; reducing fatigue; improving quality of life of both patients and caregivers
Vonk Noordegraaf et al [120], 2014	The Netherlands	Randomized controlled trial	Community, home, or retirement home	Patients—intervention: n=110; control: n=105	Gynecological surgery	Personalized eHealth program after gynecological surgery	Web app	Enhancing patient-provider communication; improving symptom management; achieving self-empowerment
Wang et al [121], 2017	China	Randomized controlled trial	Community, home, or retirement home	Patients—intervention: n=55; control: n=65	Chronic obstructive pulmonary disease	Web-based coaching program using EHRs^ac^	Web app	Enhancing patient-provider communication; increasing self-care; improving symptom management; assessing patients’ needs
Wang et al [122], 2018	China	Randomized controlled trial	Community, home, or retirement home	Patients—intervention: n=100; control: n=103	Stoma	Stoma home care mobile app	Mobile app	Enhancing psychosocial adjustment; fostering self-efficacy; assessing stoma complication incidence
Werhahn et al [123], 2019	Germany	Feasibility (pilot) study	Community, home, or retirement home	Patients—intervention: n=10	HF	CPMP^ad^	Mobile app and wearable device	Monitoring pulse, oxygen, HR, BP, and weight at home; ability to identify symptoms of HF; improving physical activity; encouraging lifestyle changes; improving symptom management; assessing patients’ needs; assessing rates of complications; medication management; monitoring physical activity (daily step count); PRO measures
Willeit et al [124], 2020	Austria	Randomized controlled trial	Community, home, or retirement home	Patients—intervention: n=1438; control: n=711	Stroke	MyStrokecard	Web app	Enhancing patient-provider communication; ability to identify symptoms of HF; enhancing patients’ knowledge, skills, and confidence; encouraging lifestyle changes; improving symptom management; improving quality of life; risk factor monitoring

^a^DHI: digital health intervention.

^b^QoC: quality of care.

^c^HF: heart failure.

^d^ACL: anterior cruciate ligament.

^e^PRO: patient-reported outcome.

^f^CPR: cardiopulmonary resuscitation.

^g^HR: heart rate.

^h^BP: blood pressure.

^i^PATH: Personal Assistant for Tracking Health.

^j^SAFER: Studying Adverse Events From Elective Surgery Research.

^k^mHealth: mobile health.

^l^SAM: Smart About Meds.

^m^OPTILOGG: home intervention system.

^n^SMMRT: Secure Messaging for Medication Reconciliation Tool.

^o^SPACE: Self-Management Program of Activity Coping and Education.

^p^HF-SMART: Heart Failure Self-Management And Readmission Prevention Technique.

^q^iADAPT: mobile health platform for strategy training in inpatient stroke rehabilitation.

^r^DPE: digital patient engagement.

^s^IGB: iGetBetter.

^t^MHAV: My Health at Vanderbilt.

^u^DTx: digital therapeutics.

^v^mVS: mHealth-supported volunteer-assisted self-help.

^w^PHA: personal health assistant.

^x^IBD: inflammatory bowel disease.

^y^NeCR: nurse-led eHealth cardiac rehabilitation.

^z^CRMP: cardiac rehabilitation maintenance program.

^aa^MMS: ManageMySurgery.

^ab^My-HF: My Hip Fracture.

^ac^EHR: electronic health record.

^ad^CPMP: cardio patient monitoring platform.

Platform-based patient-clinician DHIs were implemented using a mobile app (59/97, 61%) [28,30,31,34-36,38,41,43,45-49, 52-57,59,60,62,63,66,70,71,73,74,76,79-84,87,88,90-92, 95,96,98,100,103,104,106-108,111-117,122,123], a web-based platform (28/97, 29%) [32,33,39,40,42,44,50,51,58,61, 64,65,67,68,72,78,85,97,99,101,102,105,109,118-121,124], or a combination of both (10/97, 10%) [29,37,69,75,77,86,89,93,94,110].

**Figure 2:**
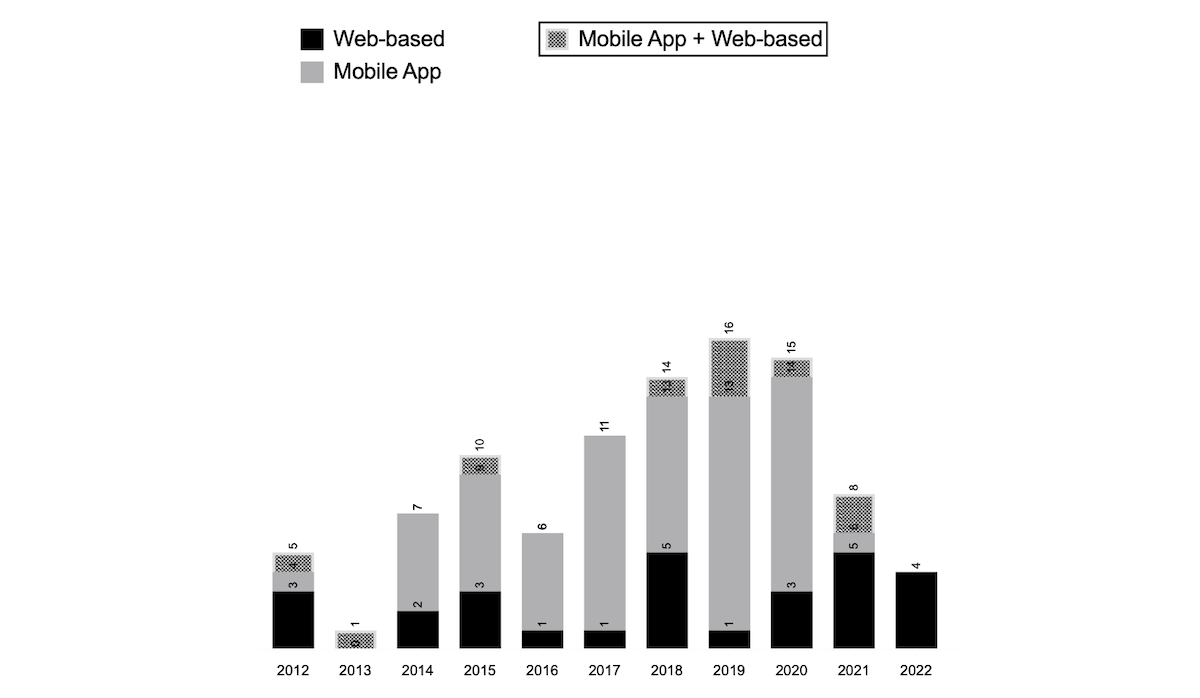
Frequency distribution of platform-based digital health interventions used for care transitions by year.

Figure 2 illustrates the frequency distribution of platform-based DHIs by year of publication.

Only 5% (5/97) of the studies [83,91,96,109,121] included all 3 types of continuity of care [11]. Informational continuity was frequently implemented through patient education and facilitating patient-provider communication. The most common ways in which management continuity was implemented was providing an assessment of the patient, monitoring the patient’s health status after discharge, and facilitating follow-up care. Relational continuity was the least implemented, with only 6% (6/97) of the studies in which the interventions included counseling or rapport building [50,82,91,96,109,121]. A more detailed description of each of the interventions using the Template for Intervention Description and Replication [26] is available in Multimedia Appendix 4 [28-124].

### Outcome Measures

The included studies reported various outcomes measures. The most common were grouped as health care use, complications, and wellness outcomes.

#### Health Care Use

A total of 21% (20/97) of the studies reported on readmission rates [36,52-54,57,58,60,66,67,70,80,81,86,87,90,94,102, 108,111,118]. Of the 20 studies, only 3 (15%) [58,102,108] showed a significant improvement, 10 (50%) reported their results as nonsignificant [36,52,60,67,80,81,86,90,94,118], 1 (5%) reported mixed results [66], and 6 (30%) [53,54,57,70,87,111] did not perform a statistical test.

In total, 8% (8/97) of the studies [36,52,60,80,81,94,108,118] reported on emergency department visits. Of the 8 studies, 2 (25%) [36,108] showed a significant improvement, whereas the remaining 6 (75%) showed no significant improvements.

#### Complications

A total of 8 studies [28,37,52,53,61,80,81,91] reported on complication rates, and only 1 (12%) [91] showed significant improvement in the complication rates compared to the control group; 3 (38%) [52,80,81] showed nonsignificant results, whereas the other 4 (50%) [28,37,53,61] did not perform a statistical test (Table 2).

**Table 2:** Studies reporting on health care use and complications.

Study	Name of DHI^a^	Participants	Readmission	ED^b^ visits	Complications	Direction and magnitude of effect
Agri et al [28], 2020	Maela	Intervention: n=43	—^c^	—	NA^d^	Among the 43 patients, the app detected 12 adverse events, and 10 (83%) were handled through the app.
Ben-Ali et al [36], 2021	SeamlessMD	Intervention: n=1108	NS^e^	Significant	—	ED visits: negative, *P*=.03, and magnitude not reported; 30-day readmissions: negative, NS, and magnitude not reported
Birkhäuser et al [37], 2020	Cellphone-based health care app	Intervention: n=18	—	—	NA	In total, 2 patients required readmission within the study period of 90 days because of postoperative complications.
Eustache et al [52], 2023	Same-day discharge mHealth^f^ app (CareSense)	Intervention: n=94; control: n=256	NS	NS	NS	30-day complications: negative, *P*=.18, and magnitude not reported; 30-day ED visits: no difference and *P*=.59; readmissions: positive, *P*=.35, and magnitude not reported
Felbaum et al [53], 2018	TrackMyRecovery	Intervention: n=56	NA	—	NA	There was 1 postoperative complication. There were no readmissions.
Ganapathy et al [54], 2017	Patient Buddy	Patients: n=40; caregivers: n=40	NA	—	—	A total of 17 patients (42.5%) were readmitted within 30 days.
Habib et al [57], 2021	Medication adherence mobile app	Intervention: n=23; control: n=26	NA	—	—	Hospital readmissions: negative (8.7% for the intervention group vs 15.4% for the control group); ED visits: positive (21.7% for the intervention group vs 19.2% for the control group)
Hägglund et al [58], 2015	Home intervention system (OPTILOGG^g^)	Intervention: n=32; control: n=40	Significant	—	—	HF^h^-related days in hospital (readmissions): negative, *P*<.005, and magnitude not reported
Heuser et al [60], 2021	SeamlessMD	Intervention: n=396; control: n=458	NS	NS	—	ED visits without subsequent readmission: no difference and *P*=.65; ED visits with subsequent readmission: no difference and *P*=.99; readmissions: no difference and *P*=.97
Heyworth et al [61], 2014	SMMRT^i^	Intervention: n=60	—	—	NA	23 potential adverse drug events observed
Indraratna et al [66], 2022	TeleClinical Care	Intervention: n=81; control: n=83	Mixed	—	—	Unplanned 30-day readmissions: no difference, *P*=.97, and magnitude not reported; total readmissions at 6 months: negative, *P*=.02, and magnitude not reported; cardiac readmissions at 6 months: negative, *P*=.03, and magnitude not reported
Johnson et al [67], 2022	HF-SMART^j^	Intervention: n=16; control: n=15	NS	—	—	30-day readmissions: positive, *P*=.65, and magnitude not reported; 90-day readmissions: positive, *P*=.70, and magnitude not reported
Keng et al [70], 2020	Home to Stay digital program after colorectal surgery	Intervention: n=106	NA	—	—	The 30-day readmission rate was 6% and lower than the 30-day readmission rate of 18% reported for the 4 months before the start of the study.
Lee et al [80], 2022	Mobile app follow-up for same-day discharge	Intervention: n=48; control: n=73	NS	NS	NS	30-day complications: positive, *P*=.81, and magnitude not reported; 30-day ED visits: positive, *P*=.66, and magnitude not reported; 30-day readmissions: positive, *P*=.68, and magnitude not reported
Lee et al [81], 2022	mHealth remote postdischarge monitoring	Intervention: n=70; control: n=35	NS	NS	NS	ED visits: no difference and *P*>.99; readmissions: negative, *P*=.37, and magnitude not reported; incidence of 30-day complications: negative, *P*=.58, and magnitude not reported
María Gómez et al [86], 2022	mHealth app for patients with type 2 diabetes transitioning from inpatient to outpatient care	Intervention: n=41; control: n=45	NS	—	—	Hospitalization for diabetes: negative, *P*=.06, and magnitude not reported
Marvel et al [87], 2021	Acute myocardial infarction DHI	Intervention: n=200; control: n=864	NA	—	—	Risk of readmission within 30 days after discharge: negative, *P*=.02, and magnitude not reported
Paruchuri et al [90], 2021	Wellframe	Intervention: n=118; historical control group: n=343	NS	—	—	All-cause readmission within 30 days: negative, *P*=.70, and magnitude not reported; all-cause readmission within 90 days: negative, *P*=.39, and magnitude not reported
Peng et al [91], 2022	Mobile continuous nursing platform	Intervention: n=47; control: n=47	—	—	Significant	Total complication rates: negative, *P*<.05, and magnitude not reported
Pooni et al [94], 2022	Home to Stay app	Intervention: n=128; control: n=125	NS	NS	—	30-day ED visits: negative, *P*=.49, and magnitude not reported; 30-day readmissions: negative, *P*=.55, and magnitude not reported
Schenkel et al [102], 2020	ActiCare	Intervention: n=28; control: n=28	Significant	—	—	Hospital readmissions (events): negative, *P*<.001, and magnitude not reported
Stapler et al [108], 2022	St. Joe’s Health App	Patients (preintervention group: n=1052; postintervention group: n=668)	Significant	Significant	—	Readmissions: negative, *P*<.001, and magnitude not reported; ED visit rate: negative, *P*<.001, and magnitude not reported
Symer et al [111], 2017	Gastrointestinal mHealth app	Intervention: n=31	NA	—	—	One patient was readmitted.
Visperas et al [118], 2021	JointCOACH	Intervention: n=204; control: n=195	NS	NS	—	ED visits: no difference and NS; readmissions: no difference and NS

^a^DHI: digital health intervention.

^b^ED: emergency department.

^c^Missing data or not applicable.

^d^NA: not available.

^e^NS: nonsignificant.

^f^mHealth: mobile health.

^g^OPTILOGG home intervention system.

^h^HF: heart failure.

^i^SMMRT: Secure Messaging for Medication Reconciliation Tool.

^j^HF-SMART: Heart Failure Self-Management And Readmission Prevention Technique.

#### Wellness Outcomes

A total of 14% (14/97) of the studies reported on quality of life [31,45,48,50,59,65,67,69,82,85,91,97,109,120]. Of the 14 studies, 8 (57%) [48,50,65,69,85,91,97,120] showed a significant improvement, whereas the other 6 (43%) reported their results to be nonsignificant. In total, 8 studies [31,51,68,76,82,91,109,115] were conducted on self-care, and 4 (50%) [82,91,109,115] showed significant results. In addition, 6 studies [31,51,65,94,119,122] reported on mental health outcomes, with 4 (67%) [65,94,119,122] showing significant improvements. A total of 8 studies [29,41,42,50,97,109,114,123] were identified for physical activity, with 5 (62%) [41,97,109,114,123] showing significant results (Table 3).

**Table 3:** Wellness outcomes.

Study	Name of DHI^a^	Participants	Quality of life	Self-care	Mental health	Physical activity	Direction and magnitude of effect
Antypas and Wangberg [29], 2014	Internet- and mobile-based tailored intervention to enhance maintenance of physical activity after cardiac rehabilitation	Intervention: n=7; control: n=12	— ^b^	—	—	Mixed	Physical activity 1 month after discharge: positive, Kolmogorov-Smirnov *Z*=0.823, and *P*=.38; physical activity 3 months after discharge: positive, Kolmogorov-Smirnov *Z*=1.397, and *P*=.02
Athilingam et al [31], 2017	Mobile app to improve self-care behaviors and quality of life for patients with HF^c^	Intervention: n=9; control: n=90	NS^d^	Mixed	NS	—	Self-care maintenance: positive, t_11_=0.083 and *P*=.93; self-care management: positive, t_11_=3.38 and *P*=.01; self-care confidence: positive, t_11_=2.53 and *P*=.28; depression: negative, t_11_=1.97 and *P*=.07; quality of life: negative, t_11_=–1.43 and *P*=.18
Cheng et al [41], 2022	Mobile app for home-based rehabilitation after hip fracture	Intervention: n=19; control: n=20	—	—	—	Significant	First-month exercise adherence: positive, *P*=.03, and magnitude not reported
Cox et al [42], 2015	ActivOnline	Intervention: n=10	—	—	—	NA^e^	Participants recorded a mean of 35 (range 15-57) physical activity sessions during the intervention period, equating to a mean of 4 recorded sessions of physical activity each week.
De Batlle et al [45], 2021	CONNECARE	Intervention: n=48; control: n=28	NS	—	—	—	Quality of life (SF-12^f^): positive, *P*=.10, and magnitude not reported
Devito Dabbs et al [48], 2016	Pocket PATH^g^	Intervention: n=99; control: n=102	Significant	—	—	—	Self-care: positive, group effect size=1.67, and *P*=.59
Duan et al [50], 2018	Health behavior intervention for patients with coronary heart disease through the web	Intervention: n=44; control: n=39	Significant	—	—	NS	Quality of life: positive, *F*_1, 79_=16.36, and *P*<.001; physical activity: positive, *F*_1, 81_=1.33, and *P*=.25
Dukeshire et al [51], 2012	Website tailored to women recovering at home after hysterectomy	Intervention: n=31	—	—	NA	—	The website reduced anxiety and worry for patients.
Heiney et al [59], 2020	Healthy Heart	Intervention: n=12	NS	NS	—	—	Quality of life: positive, *P*=.15, and magnitude not reported; Self-Care of Heart Failure Index—maintenance: difference score=9.37 and *P*=.15; Self-Care of Heart Failure Index—management: difference score=15.00 and not applicable (presample too small); Self-Care of Heart Failure Index—confidence: difference score=7.04 and *P*=.17
İlaslan and Özer [65], 2022	Web-based training and follow-up for patients with HF	Intervention: n=32; control: n=32	Significant	—	Significant	—	Quality of life—LVD-36^h^: negative, *F*=77.01, and *P*<.001
Johnson et al [67], 2022	HF-SMART^i^	Intervention: n=16; control: n=15	NS	—	—	—	Quality of life at 30 days: positive, *P*=.09, and magnitude not reported; quality of life at 90 days; negative, *P*=.10, and magnitude not reported
Kang et al [68], 2022	Web-based discharge education program	Intervention: n=43; control: n=42	—	NS	—	—	Self-care ability over time: positive, *F*_1, 60_=8.934, and *P*=.004 (significant); self-care ability—group and time interaction: positive, *F*_1, 60_=3.007, and *P*=.09
Kargar et al [69], 2020	Self-care educational mobile app for burns	Intervention: n=30; control: n=30	Significant	—	—	—	Quality of life: positive, *P*<.001, and magnitude not reported
Kooij et al [76], 2021	Self-management app for high-risk patients with chronic obstructive pulmonary disease	Patients: n=39	—	NS	—	—	Self-management knowledge and coping: positive, *P*=.75, and magnitude not reported
Liu et al [82], 2021	Together	Intervention: n=49; control: n=49	NS	Significant	—	—	Self-efficacy: positive, *F*=8.506, and *P*=.004; quality of life: positive, *F*=0.082, and *P*=.78
Lyu et al [85], 2021	Nurse-led web-based transitional care program	Intervention: n=58; control: n=58	Significant	—	—	—	Quality of life: positive, *d*=0.52, and *P*<.01; self-efficacy: positive, *d*=0.50, and *P*<.01
Peng et al [91], 2022	Mobile continuous nursing platform	Intervention: n=47; control: n=47	Significant	Significant	—	—	Self-care ability: positive, *P*<.05, and magnitude not reported; quality of life (SF-36^j^): positive, *P*<.05, and magnitude not reported
Pooni et al [94], 2022	Postdischarge Home to Stay mobile app	Intervention: n=128; control: n=125	—	—	Significant	—	Feeling worried or anxious: negative and *P*<.001
Reid et al [97], 2012	CardioFit internet-based expert system	Intervention: n=115; control: n=118	Significant	—	—	Significant	Pedometer-measured steps per day: positive, *F*=5.226, and *P*=.02; heart disease health-related quality of Life (27-item MacNew instrument): positive, *F*=1.785, and *P*=.11
Su and Yu [109], 2021	NeCR^k^ system	Intervention: n=73; control: n=73	NS	Significant	—	Significant	Mean daily steps 6 weeks after the intervention: positive, *P*=.02, and magnitude not reported; mean daily steps 12 weeks after the intervention: positive, *P*=.006, and magnitude not reported; self-efficacy: positive, *P*=.005, and magnitude not reported; MacNew health-related quality of life, positive, *P*=.06, and magnitude not reported
Torri et al [114], 2018	CRMP^l^	Intervention: n=26; control: n=27	—	—	—	Significant	Self-reported physical activity: positive, *P*=.35, and magnitude not reported
Van den Berg et al [115], 2016	CARE4STROKE	Intervention: n=31; control: n=32	—	Significant	—	—	Self-efficacy: positive, *P*=.008, and magnitude not reported
Vloothuis et al [119], 2019	CARE4STROKE digital intervention	Intervention: n=32; control: n=34	—	—	Significant	—	Patient anxiety: negative, *P*=.02, and magnitude not reported; caregiver depression: negative, *P*=.003, and magnitude not reported
Vonk Noordegraaf et al [120], 2014	Personalized eHealth program after gynecological surgery	Intervention: n=110; control: n=105	Significant	—	—	—	Quality of life: positive, between-group mean total score difference=30 (95% CI 4-57), and *P*=.02 (significant)
Wang et al [122], 2018	Stoma home care mobile app	Intervention: n=100; control: n=103	—	—	Significant	—	Psychosocial adjustment (1-, 3-, and 6-month follow-ups): positive, *F*=81.21, and *P*<.001
Werhahn et al [123], 2019	CPMP^m^	Patients: n=10	—	—	—	Significant	Mean daily step count: positive, *P*<.001, and magnitude not reported

^a^DHI: digital health intervention.

^b^Missing data or not applicable.

^c^HF: heart failure.

^d^NS: nonsignificant.

^e^NA: not available.

^f^SF-12: 12-Item Short Form Health Survey.

^g^PATH: Personal Assistant for Tracking Health.

^h^LVD-36: left ventricular dysfunction questionnaire.

^i^HF-SMART: Heart Failure Self-Management And Readmission Prevention Technique.

^j^SF-36: 36-Item Short Form Health Survey.

^k^NeCR: nurse-led eHealth cardiac rehabilitation.

^l^CRMP: cardiac rehabilitation maintenance program.

^m^CPMP: cardio patient monitoring platform.

### Patient, Caregiver, and Health Care Provider Barriers

Eight unique barriers were identified in the included studies: (1) lack of interest (13/97, 13%) [28,34,56,63,64,70,76,89,95,106,107,118,124], (2) time constraints (10/97, 10%) [44,54,56,76,78,79,96,106,109,115], (3) technological issues (7/97, 7%) [41,54,62,79,88,96,111], (4) usability issues (7/97, 7%) [42,62,71,76,96,107,117], (5) language barrier (4/97, 4%) [28,66,89,96], (6) irrelevant content of DHIs (3/97, 3%) [96,106,117], (7) lack of comfort (3/97, 3%) [78,115,117], and (8) lack of support and engagement (1/97, 1%) [106].

### Patient, Caregiver, and Health Care Provider Enablers

Seven unique enablers were identified: (1) ability to use the DHI (17/97, 18%) [28,36,62,64,67,70,76,78,80,81,89, 96,99,101,111,116,117], (2) ease of use (11/97, 11%) [49,55,57,58,68,76,85,101,105,111,121], (3) ability to collaborate with patients (1/97, 1%) [44], (4) caregiver support (1/97, 1%) [115], (5) confidence in the technology (1/97, 1%) [51], (6) convenience of using the DHIs (1/97, 1%) [62], and (7) participation in the development and implementation processes (1/97, 1%) [45] (Multimedia Appendix 5 [10,28,31,33,34,36,38-42,44,45,49,51,54-59,61-68,70,71,75, 76,78-82,85,87-90,93,95,96,99,101,105-107,109,111,112,115-118,121,123,124]).

### Conflicting Themes

In total, 3 themes were identified as both barriers and enablers. Many studies (15/97, 15%) reported that patients or caregivers were limited by their access to technology or the internet (barrier) [28,36,51,54,56,61,63,70,75,90,99,103,107,111,118], whereas other studies (28/97, 29%) reported that the patients or caregivers had access to these resources (enabler) [28,39,42,51,54,58,61,64,65,67,70,75,76,79-82,90,93,95,99, 101,105,109,112,116,118,121]. Similarly, a few studies (4/97, 4%) reported that participants had difficulty with understanding the DHIs (barrier) [28,49,64,117], whereas other studies (19/97, 20%) reported that the DHIs were easy to understand (enabler) [31,33,34,38,49,55,57,58,68,76,80,81,85,87,93,101,105,111,121]. Finally, a recurring theme that acted as both a barrier (11/97, 11%) [31,36,59,63,64,89,99,101,107,112,118] and an enabler (17/97, 18%) [39,40,42,58,59,64,65,76,78,82,90,99, 101,109,116,118,123] was whether the participants had digital literacy to use the DHIs.

## Discussion

### Summary

In this scoping review, we summarized the current evidence on platform-based patient-clinician DHIs specific to hospital-to-home care transitions and the reported barriers to and enablers of the uptake and implementation of these platform-based patient-clinician DHIs.

#### Mobile Apps Versus Web-Based Platforms

Most of the included studies used either a mobile app (59/97, 61%) or a combination of a mobile app and a web-based platform (10/97, 10%). Apps are unique because they are software programs that have been developed to run on a mobile device and are tailored to achieve a specific goal [125]. This is interesting because many studies have used other digital health tools, including e-charts [126], telehealth [127], and monitoring devices [128]. This trend may illustrate the benefits of using a mobile app over other types of digital health tools. This can include convenience, such as portability; effective communication; using a point of care for many different purposes; and immediate up-to-date information, guidelines, or medical literature [129]. Mobile apps have multiple uses in health care and have demonstrated numerous benefits, such as improved accuracy of patient documentation, improved workflow patterns or efficiency, and increased productivity of health care providers [129]. More specifically, the continuity of care between the hospital and the home.

#### Effectiveness of Platform-Based DHIs

The included studies had a broad range of outcome measures, and overall, these outcomes showed mixed results. The studies on platform-based DHIs did not show a significant improvement in readmission rates (only 3/20, 15% showed significance), emergency department visits (only 2/8, 25% showed significance), or complication rates (only 1/8, 12% showed significance). However, the studies reported promising results for quality of life (8/14, 57% of the studies), self-care (4/8, 50% of the studies), mental health (4/6, 67% of the studies), and physical activity (5/8, 62% of the studies). Further research is needed to better plan and evaluate the overall effectiveness of these specific DHIs by clearly linking outcomes with specific interventions.

#### Barriers to and Enablers of the Uptake and Implementation of Platform-Based DHIs

The most prominent barriers were lack of interest and time constraints to use the DHIs, and the most prominent enablers reported were the ability to use the DHIs and their ease of use. This reveals the importance of simple, user-friendly DHIs as the patient’s confidence in using them will determine how engaged they are throughout the intervention. Another important factor that plays a role in whether DHIs will be successful is whether the patient has access to the proper technological resources. This came up as a prominent barrier if they lacked the appropriate resources or as an enabler if they possessed what they needed. This reveals an important factor when considering the implementation of DHIs as the target population must have access to the correct resources to allow the intervention to take place.

#### Strengths and Limitations

Our study had several strengths and limitations. We designed an in-depth a priori protocol. The search strategy was developed and peer reviewed by a research librarian with extensive knowledge of scoping and systematic review methodologies. This scoping review was unique in that it specifically examined platform-based patient-clinician DHIs and not all types of DHIs. This allowed us to examine interventions that implemented elements that may promote more patient engagement, foster better communication between patients and health care providers, and integrate everything needed into one convenient program. The wide variety of studies included in this review led to a wide range of outcomes. We focused on the outcomes of health care use, complications, and wellness during the transition from hospital to home. Some other outcomes were excluded as they were specific to the disease or procedure performed. In addition, this review did not limit the inclusion to one type of health condition. However, this allowed us to examine DHIs across multiple areas of research and evaluate barriers to and enablers of DHI implementation across health conditions.

We identified a substantial body of literature on platform-based DHIs and their role in supporting patient care transitions from hospital to home. Most studies (95/97, 98%) primarily focused on patients’ use of these DHIs. While patients are central to health care delivery, it is equally important to evaluate the effectiveness of platform-based DHIs from the health care providers’ perspective. If these systems are not user-friendly for providers, widespread adoption is unlikely. Therefore, a deeper understanding of how providers interact with DHIs is essential for their successful implementation.

The transition of patients from hospital to home is a critical process that must be carried out safely and efficiently. However, this process is inherently complex due to factors such as unclear provider roles, suboptimal communication, and the patient’s ability to manage their own care [130]. When transitions are not carried out effectively, patient care can be compromised, leading to negative outcomes [131]. Platform-based DHIs offer a promising solution to help streamline care during this vulnerable period, potentially improving the quality and safety of transitions. The findings of this work can inform future work on DHIs and, more specifically, the “MyPath to Home” DHI previously piloted for the population with hip fracture during their transition from hospital to home [33].

#### Conclusions

There is a lot of potential for using DHIs for care transitions; however, the specific elements that will improve patient outcomes need to be further explored. Specifically, further work is needed to involve all key stakeholders in the design, development, and implementation of these DHIs and understand their effectiveness to embed them in practice more broadly.

## Appendix

Multimedia Appendix 1 PRISMA-ScR (Preferred Reporting Items for Systematic Reviews and Meta-Analyses extension for Scoping Reviews) checklist.

Multimedia Appendix 2 Search strategies.

Multimedia Appendix 3 List of excluded studies.

Multimedia Appendix 4 Description of the interventions.

Multimedia Appendix 5 Barriers and enablers.

## Abbreviations

DHI: digital health intervention
PRISMA-ScR: Preferred Reporting Items for Systematic Reviews and Meta-Analyses extension for Scoping Reviews

## Data Availability

All data generated or analyzed during this study are included in this published article and its supplementary information files.
