# Supplementary material for: Platform-Based Patient-Clinician Digital Health Interventions for Care Transitions: Scoping Review
Source: J Med Internet Res. 2024 Dec 30;26:e55753. doi: 10.2196/55753 (PMC11729789; doi:10.2196/55753)
Supplement: Multimedia Appendix 2 [file jmir_v26i1e55753_app2.docx]

**Supplementary Table 1. Search strategies**

## Table 1a. Medline Search

| **Database:** **MEDLINE(R) ALL 1946 to July 12, 2022**  **Platform: Ovid**  **Date Searched: July 13, 2022** | | |
| --- | --- | --- |
| **#** | **Searches** | **Results** |
| 1 | hospital to home transition/ or patient discharge/ or Transitional Care/ | 37758 |
| 2 | (discharg* or post-hospital* or posthospital* or after-hospital* or Postoperat* or post-operat* or Postsurger* or post-surger* or Postsurgical* or post-surgical* or after surger* or after operat*).ti,ab,kf. | 1115293 |
| 3 | (transition* adj4 (home? or house? or community or residence* or hospital* or care or pathway* or protocol* or healthcare or navigat*)).ti,ab,kf. | 17379 |
| 4 | (home adj4 (return* or transition* or reintegrat* or re-integrat* or follow-up or followup)).ti,ab,kf. | 7364 |
| 5 | or/1-4 | 1139735 |
| 6 | Computers/ or Internet/ or Internet-Based Intervention/ or Therapy, Computer-Assisted/ or Mobile Applications/ or Web Browser/ | 148781 |
| 7 | (mhealth or m-health or "mobile health" or ehealth or e-health or digital health or ePRO).ti,ab,kf. | 23925 |
| 8 | ((web or website? or web-based or internet* or online or patient* or mobile* or computer* or PC or tablet* or digital* or digiti* or electronic*) adj3 (application* or app? or portal* or platform* or hub? or dashboard*)).ti,ab,kf. | 65838 |
| 9 | or/6-8 | 217624 |
| 10 | 5 and 9 | 5531 |
| 11 | limit 10 to yr="2011 -Current" | 3530 |

## Table 1b. Embase Search

| **Database:** **Embase Classic+Embase 1947 to 2022 July 12**  **Platform: Ovid**  **Date Searched: July 13, 2022** | | |
| --- | --- | --- |
| **#** | **Searches** | **Results** |
| 1 | exp *hospital discharge/ or *transitional care/ | 17382 |
| 2 | (discharg* or post-hospital* or posthospital* or after-hospital* or Postoperat* or post-operat* or Postsurger* or post-surger* or Postsurgical* or post-surgical* or after surger* or after operat*).ti,ab,kf. | 1669563 |
| 3 | (transition* adj4 (home? or house? or community or residence* or hospital* or care or pathway* or protocol* or healthcare or navigat*)).ti,ab,kf. | 26569 |
| 4 | (home adj4 (return* or transition* or reintegrat* or re-integrat* or follow-up or followup)).ti,ab,kf. | 12153 |
| 5 | or/1-4 | 1696535 |
| 6 | exp *personal computer/ or *computer/ or *internet/ or *web-based intervention/ or exp *computer assisted therapy/ or exp *self-care software/ or exp *mobile application/ or *web browser/ | 95259 |
| 7 | (mhealth or m-health or "mobile health" or ehealth or e-health or digital health or ePRO).ti,ab,kf. | 25577 |
| 8 | ((web or website? or web-based or internet* or online or patient* or mobile* or computer* or PC or tablet* or digital* or digiti* or electronic*) adj3 (application* or app? or portal* or platform* or hub? or dashboard*)).ti,ab,kf. | 90690 |
| 9 | or/6-8 | 191964 |
| 10 | 5 and 9 | 7981 |
| 11 | limit 10 to yr="2011 -Current" | 5904 |

## Table 1c. Cochrane Central Register of Controlled Trials Search

| **Database:** **EBM Reviews - Cochrane Central Register of Controlled Trials June 2022**  **Platform: Ovid**  **Date Searched: July 13, 2022** | | |
| --- | --- | --- |
| **#** | **Searches** | **Results** |
| 1 | hospital to home transition/ or patient discharge/ or Transitional Care/ | 1832 |
| 2 | (discharg* or post-hospital* or posthospital* or after-hospital* or Postoperat* or post-operat* or Postsurger* or post-surger* or Postsurgical* or post-surgical* or after surger* or after operat*).ti,ab,kw. | 187062 |
| 3 | (transition* adj4 (home? or house? or community or residence* or hospital* or care or pathway* or protocol* or healthcare or navigat*)).ti,ab,kw. | 1772 |
| 4 | (home adj4 (return* or transition* or reintegrat* or re-integrat* or follow-up or followup)).ti,ab,kw. | 2092 |
| 5 | or/1-4 | 189291 |
| 6 | Computers/ or Internet/ or Internet-Based Intervention/ or Therapy, Computer-Assisted/ or Mobile Applications/ or Web Browser/ | 7008 |
| 7 | (mhealth or m-health or "mobile health" or ehealth or e-health or digital health or ePRO).ti,ab,kw. | 4347 |
| 8 | ((web or website? or web-based or internet* or online or patient* or mobile* or computer* or PC or tablet* or digital* or digiti* or electronic*) adj3 (application* or app? or portal* or platform* or hub? or dashboard*)).ti,ab,kw. | 11009 |
| 9 | or/6-8 | 19912 |
| 10 | 5 and 9 | 1561 |
| 11 | limit 10 to yr="2011 -Current" | 1357 |

## Table 1d. CINAHL Search

| **Database: CINAHL**  **Platform: EBSCOHost**  **Date Searched: July 13, 2022** | | | | |
| --- | --- | --- | --- | --- |
| **#** | **Query** | **Limiters/Expanders** | **Last Run Via** | **Results** |
| S16 | S7 AND S14 | Limiters - Published Date: 20110101-20221231 Expanders - Apply related words; Apply equivalent subjects Search modes - Boolean/Phrase | Interface - EBSCOhost Research Databases Search Screen - Advanced Search Database - CINAHL | 1,961 |
| S15 | S7 AND S14 | Expanders - Apply related words; Apply equivalent subjects Search modes - Boolean/Phrase | Interface - EBSCOhost Research Databases Search Screen - Advanced Search Database - CINAHL | 2,507 |
| S14 | S8 OR S9 OR S10 OR S11 OR S12 OR S13 | Expanders - Apply related words; Apply equivalent subjects Search modes - Boolean/Phrase | Interface - EBSCOhost Research Databases Search Screen - Advanced Search Database - CINAHL | 102,916 |
| S13 | TI ( ((web or website# or web-based or internet* or online or patient* or mobile* or computer* or PC or tablet* or digital* or digiti* or electronic*) N3 (application* or app# or portal* or platform* or hub# or dashboard*)) ) OR AB ( ((web or website# or web-based or internet* or online or patient* or mobile* or computer* or PC or tablet* or digital* or digiti* or electronic*) N3 (application* or app# or portal* or platform* or hub# or dashboard*)) ) | Expanders - Apply related words; Apply equivalent subjects Search modes - Boolean/Phrase | Interface - EBSCOhost Research Databases Search Screen - Advanced Search Database - CINAHL | 23,893 |
| S12 | TI ( (mhealth or m-health or "mobile health" or ehealth or e-health or digital health or ePRO) ) OR AB ( (mhealth or m-health or "mobile health" or ehealth or e-health or digital health or ePRO) ) | Expanders - Apply related words; Apply equivalent subjects Search modes - Boolean/Phrase | Interface - EBSCOhost Research Databases Search Screen - Advanced Search Database - CINAHL | 11,773 |
| S11 | (MH "Mobile Applications") OR (MH "Web Browsers") | Expanders - Apply related words; Apply equivalent subjects Search modes - Boolean/Phrase | Interface - EBSCOhost Research Databases Search Screen - Advanced Search Database - CINAHL | 11,408 |
| S10 | (MH "Therapy, Computer Assisted") OR (MH "Drug Therapy, Computer Assisted") | Expanders - Apply related words; Apply equivalent subjects Search modes - Boolean/Phrase | Interface - EBSCOhost Research Databases Search Screen - Advanced Search Database - CINAHL | 5,977 |
| S9 | (MH "Internet") OR (MH "Internet-Based Intervention") | Expanders - Apply related words; Apply equivalent subjects Search modes - Boolean/Phrase | Interface - EBSCOhost Research Databases Search Screen - Advanced Search Database - CINAHL | 54,254 |
| S8 | (MH "Computers, Portable+") | Expanders - Apply related words; Apply equivalent subjects Search modes - Boolean/Phrase | Interface - EBSCOhost Research Databases Search Screen - Advanced Search Database - CINAHL | 9,735 |
| S7 | S1 OR S2 OR S3 OR S4 OR S5 OR S6 | Expanders - Apply related words; Apply equivalent subjects Search modes - Boolean/Phrase | Interface - EBSCOhost Research Databases Search Screen - Advanced Search Database - CINAHL | 296,445 |
| S6 | TI ( (home N4 (return* or transition* or reintegrat* or re-integrat* or follow-up or followup)) ) OR AB ( (home N4 (return* or transition* or reintegrat* or re-integrat* or follow-up or followup)) ) | Expanders - Apply related words; Apply equivalent subjects Search modes - Boolean/Phrase | Interface - EBSCOhost Research Databases Search Screen - Advanced Search Database - CINAHL | 5,349 |
| S5 | TI ( (transition* N4 (home# or house# or community or residence* or hospital* or care or pathway* or protocol* or healthcare or navigat*)) ) OR AB ( (transition* N4 (home# or house# or community or residence* or hospital* or care or pathway* or protocol* or healthcare or navigat*)) ) | Expanders - Apply related words; Apply equivalent subjects Search modes - Boolean/Phrase | Interface - EBSCOhost Research Databases Search Screen - Advanced Search Database - CINAHL | 12,743 |
| S4 | TI ( (discharg* or post-hospital* or posthospital* or after-hospital* or Postoperat* or post-operat* or Postsurger* or post-surger* or Postsurgical* or post-surgical* or after surger* or after operat*) ) OR AB ( (discharg* or post-hospital* or posthospital* or after-hospital* or Postoperat* or post-operat* or Postsurger* or post-surger* or Postsurgical* or post-surgical* or after surger* or after operat*) ) | Expanders - Apply related words; Apply equivalent subjects Search modes - Boolean/Phrase | Interface - EBSCOhost Research Databases Search Screen - Advanced Search Database - CINAHL | 276,991 |
| S3 | (MH "Transitional Care") | Expanders - Apply related words; Apply equivalent subjects Search modes - Boolean/Phrase | Interface - EBSCOhost Research Databases Search Screen - Advanced Search Database - CINAHL | 3,103 |
| S2 | (MH "Patient Discharge") OR (MH "Patient Discharge Education") | Expanders - Apply related words; Apply equivalent subjects Search modes - Boolean/Phrase | Interface - EBSCOhost Research Databases Search Screen - Advanced Search Database - CINAHL | 23,708 |
| S1 | (MH "Hospital to Home Transition") | Expanders - Apply related words; Apply equivalent subjects Search modes - Boolean/Phrase | Interface - EBSCOhost Research Databases Search Screen - Advanced Search Database - CINAHL | 56 |
