# Supplementary material for: Platform-Based Patient-Clinician Digital Health Interventions for Care Transitions: Scoping Review
Source: J Med Internet Res. 2024 Dec 30;26:e55753. doi: 10.2196/55753 (PMC11729789; doi:10.2196/55753)
Supplement: Multimedia Appendix 4 [file jmir_v26i1e55753_app4.docx]

**Supplementary Table 3. Description of interventions (using TIDier Checklist [26])**

| **Lead Author (year)** | **Name of DHIs** | **WHY?** | **WHAT?** | **WHO ?** | **HOW ?** | **WHERE?** | **WHEN?** | **HOW MUCH?** | **Tailoring /modification** | **HOW WELL?** |
| --- | --- | --- | --- | --- | --- | --- | --- | --- | --- | --- |
| Agri (2020) [28] | Maela | The means to target shorter hospital stay include information technology strategies to improve communication between caregivers and patients in order to limit potentially avoidable readmissions. | The connected follow-up offered continuous enhanced recovery after surgery (ERAS) information delivery to the patient, as well as daily structured questionnaires. In addition, it was possible to transmit body temperature measurements and photos of the wound. | Nurse, Surgeon | Mobile app | Community/Home/Retirement home | 7 days post discharge | Once daily | Depending on the patient’s responses, alerts were generated and sent by email, short messages (SMS) or directly to the healthcare provider app, according to the predefined wishes of the user. | Compliance with the enhanced recovery after surgery (ERAS) pathway was 80% in the connected group. The connected patients completed all seven questionnaires in 72% (31/43) of cases. |
| Antypas (2014) [29] | Internet- and Mobile-Based Tailored Intervention to Enhance Maintenance of Physical Activity After Cardiac Rehabilitation | To assess the effect of a longitudinally tailored Internet- and mobile-based intervention for physical activity as an extension of a face-to-face cardiac rehabilitation stay. | Not reported for intervention group  Control group: Information about diet, physical activity, smoking, and medication, as well as online discussion forums about cardiovascular disease | Not reported | Mobile app, Web-based application | Community/Home/Retirement home | 90 days post discharge | Not reported | In some cases, participants received tailored messages via email or SMS, and were asked to plan training activities or set weekly goals based on their change stage. | Kaplan-Meier survival curves were used to analyze website adherence. Attrition rates were high. Dropout rates were higher in the tailored group than in the control group at the beginning of the intervention. |
| Armstrong (2017) [30] | QoC Health Inc mobile app | Our study builds on preexisting data by determining whether receiving follow-up via the mobile app can avert the need for in-person follow-up care. | The QoC Health Inc mobile app allows patients to submit photographs and answers to a validated quality of recovery questionnaire and a pain visual analog scale using a mobile de- vice for the first 30 days after the operation. Surgeons are able to follow patient reports on a web portal. | Primary care physician | Mobile app | Community/Home/Retirement home | 30 days post discharge | Daily monitoring for 2 weeks and then weekly monitoring for the remaining 2 weeks | The surgeon used a wireless interface to access patients data and monitor the patient’s condition (not in real time). Any red flags (abnormally high pain scores or abnormally low quality of recovery 9-item questionnaire scores) prompted in-person follow-up. | A total of 65 patients were enrolled in the study: 33 completed in-person follow-up care and 32 completed mobile app follow-up care. No patients were lost to follow-up. There were no missing data. |
| Athilingam (2017) [31] | Mobile app to improve self-care behaviors and quality of life for patients with HF | To test the feasibility of a newly developed mobile app (HeartMapp) in improving self-care behaviors and quality of life of patients with HF and to calculate effect sizes for sample size calculation for a larger study | Daily weight, symptom assessment, customized alerts, vital sign monitoring with BioHarness-3 chest strap, HF education, and walking and breathing exercises. | Nurse | Mobile app | Community/Home/Retirement home | 30 days post discharge | Daily for a total of 4 weeks | Tailored automated reminder to check weight and complete symptoms assessment | Medication adherence assessed using the Morisky Medication Adherence Questionnaire, which has eight items. |
| Avci (2018) [32] | Supportive website for Stroke Patients’ Caregivers after Discharge | To determine the frequency of stroke patients’ caregivers to utilize the supportive web site after their patients are discharged | Stroke definition, stroke course, nutrition, respiration, urinary system, bedsores, body hygiene, patient safety, medications, sleeping patterns, pain, communication with the patient, and institutions that provide assistance with care (home care centers and public institutions).  Videos include breathing exercises, postural drainage, oral care, catheter care, perineal care, supine, side-down, prone, position to side-down position, passing from side-down to prone position, pressure points in side-down position, and arm and leg exercises. | Nurse | Web-based application | Community/Home/Retirement home | 365 days post discharge | Daily use recommended | Not reported | Adherence not reported.  198 enrolled, 9 caregivers died, 42 caregivers did not use the web site |
| Backman (2020) [33] | MyPath to Home Web-Based Application for the Geriatric Rehabilitation | To manage the personalized needs of geriatric rehabilitation patients during their transition from the hospital to home. | App supports multichannel communications among patients, caregivers, and clinicians. | Social worker, Physiotherapy, Occupational therapist, Nurse, Primary care physician | Mobile app, Web-based application | Community/Home/Retirement home | 30 days post discharge | Not reported | Application designed to provide tailored discharge and care transition information | Not reported |
| Backer (2021) [34] | GenuSport | The GenuSport mobile app was designed to be easy to use for improving postoperative quadriceps weakness and knee motion. The ease of use can improve patients' compliance, and encourage them to perform regular exercises at home. | The app-based program consists of a knee trainer and an app called GenuSport. The knee trainer (GenuSport) contains three pressure sensors that are placed in the back of the knee while the patient is lying in a supine position with a 45° angle pillow elevating upper part of the body while holding the tablet with both hands. During training, the knee is in neutral rotation without lifting the hip. The patient has to engage with different modes, either pushing the knee onto the sensor or lifting in the air for an indicated amount of time. The strength measured is then transmitted via Bluetooth to the app for continuous and real-time visual feedback. | Surgeon | Mobile app | Community/Home/Retirement home | 42 days post discharge | 3-5 times daily | At the end of each training session, patients can analyze their performance. | In total, sixty patients gave consent for inclusion in the prospective randomized trial, but only 35 patients (58.3%) followed up and were included in the trial. Of those included 20 patients (60.6%; initially n=33) were randomized to the app group and the remaining 15 patients randomized to the control group (55.5%;initially n=27). |
| Bauwens (2022) [35] | Doct-Up | In France in the spring of 2020, the lockdown mandated due to the COVID-19 pandemic prevented patients from seeing their physiotherapists for 2 months. Using a self-rehabilitation smartphone app limits the negative effects of not receiving physiotherapist rehabilitation after anterior cruciate ligament (ACL) reconstruction. | The app has two parts, for the pre-operative and post-operative periods, respectively. The pre-operative part sends notifications to support the patients in their daily activities until the surgery. The post-operative part consists in video tutorials that change over time based on the advances made by the patient, pain level, and any other sources of discomfort. The app gives advice and sets daily targets. | Not reported | Mobile app | Community/Home/Retirement home | 42 days post discharge | Once daily | The post-operative part consists in video tutorials that change over time based on the advances made by the patient, pain level, and any other sources of discomfort. | Not reported |
| Ben-Ali (2021) [36] | SeamlessMD (Toronto, ON, Canada) | Improve postoperative follow-up period (enhance communication, monitoring and maintaining quality of recovery) by placing patient voices and patient involvement at the forefront of health care delivery. | It was intended to track health status, protocol compliance, and patient-reported outcomes in the postoperative period. In addition, it provides patient preoperative education, preoperative and postoperative tasks based on reminders, to-do lists, and evidence-based content. The patient had the capacity to send pictures of their wounds through the app. | Nurse, Health care team | Mobile app | Community/Home/Retirement home | 28 days post discharge | Once daily | Low-risk issues were directed to self-care education. Higher risk issues were escalated to the care team (emergency room visit recommendation or phone call to the nurse) | 66% of patients activated their app (730 of 1,108) |
| Birkhauser (2020) [37] | Cellphone-based health care application | To intensify postoperative patient-physician communication via a cellphone-based health care application (CHA) and to evaluate its potential for early detection of complications. | Individual data entry on body weight in kilograms, temperature in degrees Celsius, and fluid intake and output in liters. Patients had the option to enter individual remarks or questions on each day of data collection, and to record incisional wound healing with the photo-capture function. | Surgeon | Mobile app, Web-based application | Community/Home/Retirement home | 90 days post discharge | Automatic push notifications were generated twice a week. Between postoperative days 31 and 90, these were reduced to once a week | Patient data entry and response was specific to each patient | Not reported |
| Blewer (2020) [38] | mApp CPR training application | To facilitate cardiopulmonary resuscitation (CPR) training for family members of high-risk cardiac patients using mobile application-based (mApp). | A video of an inflatable head/torso manikin and a 22-minute instructional training.  Additionally a CPR refresher video, a practice module including elements of gamified learning, opportunity to discuss CPR with the study team, and links to CPR instructional resources | Nurse | Mobile app | Community/Home/Retirement home | Not reported | Not reported | Not reported | 784 lost to follow-up at 6months. Adherence not reported |
| Bouwsma (2018) [39] | eHealth intervention | Aimed at preventing unnecessary prolonged recovery following gynaecological surgery and preventing delayed return to work. | The interactive web portal facilitated self-management by providing patients with individual tailored convalescence recommendations throughout the entire surgical pathway as well as monitoring recovery postoperatively through an interactive self-assessment tool. Second, for those patients at risk of prolonged sick leave, a care manager was avail- able to provide additional guidance in the process of resuming work activities (occupational intervention). | Health Care Professional | Web-based application | Community/Home/Retirement home | 364 days post discharge | Each month, the patient was asked to report service use over the previous month. (reported in cost terms) | Care managers were trained to help patients identify possible barriers to resuming work activities and could assist in the planning and execution of work resumption. The interactive web portal provided patients with individual tailored convalescence recommendations throughout the entire surgical pathway. | Not reported |
| Bouwsma (2018) [40] | eHealth Intervention | Increase knowledge on appropriate postoperative recovery, improve self-management and appropriate recovery behaviour. Aimed at reducing unnecessary prolonged postoperative recovery and high societal costs. | An interactive web portal facilitated self-management through the entire surgical pathway, by providing individual tailored convalescence advice preoperatively. Postoperatively, the web portal contained an interactive self-assessment tool to monitor recovery. | Nurse, Primary care physician | Web-based application | Community/Home/Retirement home | Patients were advised to resume their work activi- ties gradually to reach full work by 2 - 6 weeks based on surgery type. | Not reported | Web portal provided individually tailored convalescence advice preoperatively. | In the intervention group, the majority of patients logged in to the web portal at least once (215/227; 94.7%). |
| Cheng (2022) [41] | Home-based rehabilitation mobile app | The study's primary hypothesis is that the home-based rehabilitation program delivered using mobile app will result in better functional outcomes compared to usual care delivered using the conventional paper handouts. Secondary hypothesis is that mobile app intervention will result in better adherence and lesser caregiver burden. | The features of the mobile app include the following: (1) Exercise program for the geriatric hip fracture patient; (2) Progress summary in a calendar format; (3) Push reminder to alert the participants and sends out motivational message; (4) Rehab knowledge (the related surgical intervention, post-operative management and precautions); (5) Video libraries of a wide range of practical caregiver skills and information; (6) Support information. | Physiotherapy | Mobile app | Community/Home/Retirement home | 60 days post discharge | Once per day (20 min - 30 min) | Participants were instructed to perform the prescribed level of exercises once per day with the course length varying from 20 min to 30min. The progression level of the exercise pro- gram was reassured by the domiciliary physiotherapists during weekly home visits based on clinical performance of the participants. | A total of 50 participants were enrolled, with 19 participants in the experimental group and 20 participants in the control group. Eleven participants had withdrawn from the study. The experimental group showed higher exercise adherence than the control group in first month (Median: 100% vs 75%; p=0.03*). |
| Cox (2015) [42] | ActivOnline | Internet-based programs may help overcome barriers to exercise reported by participants attending hospital-based exercise programs (e.g., transport and time) and may aid in improving exercise program adherence. In cystic fibrosis, routine physical activity participation may improve exercise tolerance and relieve breathlessness, as well as positively influence bone accretion, blood glucose control, and clearance of pulmonary secretions. | The program provided users with real-time graphical representation of their physical activity entries (in particular, activity duration and daily step count), as well as a written display of previous entries. There was an inbuilt messaging system for direct communication between subjects and the research team, and subjects received an automated e-mail message reminding them to log into the program when >3 days had elapsed since recording the previous entry. | Physiotherapy, research team | Web-based application | Community/Home/Retirement home | 56 days post discharge | Not pre-determined | All subjects received a telephone consultation before using ActivOnline and each fortnight from a physiotherapist experienced in the management of CF. The aim of the telephone consultation was to foster goal setting by discussing barriers and facilitators to physical activity and building motivation for physical activity participation. | Each subject logged into ActivOnline a mean of 13 +/- 11 times over the 8-week intervention period. |
| Davis (2020) [43] | Force Therapeutics | Physical therapy is commonly used to help achieving goals after total shoulder arthroplasty such as relieving pain, improving range of motion, and restoring function. Recent evidence has pointed to the success and safety of a purely physician-guided, home-based or internet-based, program versus the traditional therapist guided program. | The online portal provides pictures and videos describing how each exercise is to be performed. Each time the patient logs-in and views an exercise it is recorded in the system to help monitor their progress. They are encouraged to ask questions to their nurse navigator or their surgeon within the system. | Nurse, Surgeon | Mobile app | Community/Home/Retirement home | 102 days post discharge | Once daily | Patients interact with the program throughout the pre-operative and post-operative periods and can also interact with a nurse navigator as well as the surgeon through the online portal. | The average compliance of logins during the perioperative period was 23.1% (range 0 – 86) and compliance of tasks completed per login was 66.0% (range 0 – 100). Compliance of logins is the number of times a patient logged into the system compared to the total number of login days available to them. Compliance of tasks is the number of tasks completed out of the total number prescribed. |
| Davoody (2016) [44] | eHealth for Post-Discharge Stroke | To assess the usefulness of a stroke planning tool | Focus group assessments of the care professionals’ perceived usefulness of the prototype and use of it. | Physiotherapy, Occupational therapist, Speech therapist, Counselor | Web-based application | Community/Home/Retirement home | Not reported | Not reported | Not reported | Not reported |
| De Batlle (2021) [45] | CONNECARE | The mHealth-enabled integrated care model was implemented for the community-based prevention of unplanned hospital-related events in complex chronic patients with a high risk for hospitalization. | The self-management app consists of status and performance reports, a virtual coach with customizable automated feedback, and full communication with the care team. Additionally, a Fitbit Flex 2 digital activity tracker and any additional sensor deemed necessary by the care team, including a digital pulse-oximeter, digital scale, and digital blood pressure monitor, were fully integrated into the self-management app. | Social worker, Primary care physician, family physicians, case manager | Mobile app, Wearable device | Community/Home/Retirement home | 90 days post discharge | Not reported | A patient profile in the web-based platform accessible to all members of the care team was used for coordination and communication among professionals in the different settings, to contact the patient when needed; and assignment of a case manager in charge of supervising the whole process. | After excluding patients not meeting the inclusion criteria, 52 patients were recruited for the mHealth-enabled integrated care arm and 35 patients were recruited for the usual care arm. Final analyses were based on 48 integrated care and 28 control patients completing the follow up. |
| Debono (2016) [46] | Cornebarrieu-France | To reduce hospitalization time, care management must be patient oriented with an up-to-date monitoring structure based on recently introduced eHealth technology. Although the value of an application used to monitor various pathologies has previously been reported, this is the first study to report on its practical daily use in France for ambulatory lumbar discectomy patients. | The Mobile app recovery indicators included a visual analogue scale (VAS) for pain and a questionnaire regarding: body temperature, painful voiding disorder, motor disorder or a blood stain on the dressing. The patient-interface has a checklist which sends an alarm to the fast-tracking-unit with different color codes based on the information entered by the patient. | Nurse, Surgeon | Mobile app | Community/Home/Retirement home | 15 days post discharge | The frequency of use is unlimited during 15 days. | The patient-interface has a checklist which sends an alarm to the fast-tracking-unit with different color codes based on the information entered by the patient. | All of the 60 patients completed the phone interview at 3 months postoperatively. |
| Debono (2019) [47] | e-fitback | The first goal of Enhanced Recovery After Surgery (ERAS) is the improvement of surgical outcomes and patient experience, with an ultimate impact on a reduction in the length of stay (LOS). | The mobile app recovery indicators included a visual analog scale (VAS) for pain and a questionnaire regarding body temperature, a painful voiding disorder, a motor disorder, or a blood stain on the dressing. | Primary care physician | Mobile app | Community/Home/Retirement home | 15 days post discharge | Patients must validate the checklist at least once during the first postoperative 48 hours; after this the frequency of use is unlimited during the 15 days. | The patient interface has a checklist that sends an alarm to the Enhanced Recovery After Surgery (ERAS) team with different color codes based on the information entered by the patient. | Not reported |
| DeVito Dabbs (2016) [48] | Pocket Personal Assistant for Tracking Health (Pocket PATH") | Prevention and detection of early complications are known to reduce the likelihood of future impairments in lung function and, therefore, morbidity and mortality. The importance of self-management to promote better health outcomes after lung transplantation is well recognized. The connectivity, capabilities, and widespread use of mobile devices make mobile health (mHealth) technologies suitable for interventions that promote adherence, real-time data collection for self-monitoring, and self-management. | Pocket PATH programs allowed patients to record daily health indicators, view graphical displays of trends, and receive automatic feedback messages advising them to notify the transplant coordinator if health indicators were critical (outside the pre established parameters). | transplant coordinator | Mobile app | Community/Home/Retirement home | 365 days post discharge | Once daily | The Pocket PATH group received a smartphone with custom Pocket PATH programs to record daily health indicators, view graphical displays of trends, and receive automatic feedback messages advising them to notify the transplant coordinator if health indicators were critical (outside the preestablished parameters). | Adjusting for time and the covariates, the Pocket PATH group was more likely to show high adherence than was the usual care group (OR 1.64, 95% CI 1.01–2.66, p = 0.046). In both groups, there was a significant time effect, and the percentage of the groups who had higher adherence decreased over time. |
| Dorsch (2021) [49] | ManageHF4Life | The objective of the mobile app intervention was to improve self-monitoring, which can enhance self-management and improve health-related quality of life in heart failure. Failure to recognize clinical worsening is related to poor self-management, and one of the most common causes of heart failure readmission. | The app prompted active daily self-monitoring, provided a health status indicator to promote self-management, and included standard education on heart failure. Heart failure care includes daily monitoring of weight and symptoms, taking medications as prescribed, adhering to a low-sodium diet, and assessing changes in symptoms related to self-monitoring. | Nurse, Primary care physician | Mobile app, a Fitbit (Fitbit Inc) physical activity monitor (Fitbit Charge 2) and scale (Fitbit Aria and Aria 2). | Community/Home/Retirement home | 84 days post discharge | Once daily | The health status indicator was a stoplight (green= stable, yellow and red= worsening) and was generated from a rule-based model created by the investigators. The text below the health status indicator changed based on the color, with recommendations on self-management. | The median number of days during which the app group performed self-monitoring within the app was 63 (IQR 28-84) of the 84 days (75%). |
| Duan (2018) [50] | Health Behavior Intervention for Coronary Heart Disease Patients Through the Web | To evaluate the effect of an 8-week Web-based intervention in terms of physical activity (PA), fruit and vegetable consumption (FVC), lifestyle changes, social-cognitive outcomes, and health outcomes compared with a waiting control group in Chinese cardiac patients. | Risk perception. outcome expectancies, and goal setting; Development of action plans; Revision and adjustment of previous action plans and development of coping plans; Revision and adjustment of previous coping plans and development of behavior-specific social support. | Nurse, Primary care physician | Web-based application | Community/Home/Retirement home | 56 days post discharge | Sessions provided 4 weeks at a time | Not reported | In the IG, 44 out of 60 patients (73%) adhered to the entire 8-week intervention. |
| Dukeshire (2012) [51] | Web site tailored to women recovering at home after hysterectomy | To use the SAFER project to provide access to information on a Web site tailored to women recovering at home after hysterectomy | Information specific to the day of recovery A 2-minute checkup with detailed symptom-specific information provided for any positive replies. A free-text recovery journal General information pertaining to presurgical and postsurgical care. | Nurse, Surgeon | Web-based application | Community/Home/Retirement home | 42 days post discharge | Once a day for the first week after discharge and twice a week for the next 3 weeks thereafter | The Web site provides timely and relevant information tailored to the patient's postsurgical recovery stage. | Not reported |
| Eustache (2023) [52] | Same Day Discharge mHealth app (ACTUALLY: CareSense mobile app) | We hypothesized that a mobile app for post-discharge monitoring with patient- provider communication ability would reduce emergency visits after elective abdominopelvic colorectal surgery. | The app included patient education material on the expected postoperative course, daily questionnaires assessing post- discharge recovery, and patient-provider chat function. | Care coordinator, treating surgeon | Mobile app | Community/Home/Retirement home | 14 days post discharge | “Daily Health Check” sent daily until 7 days after discharge, and then every other day until 14 days after discharge. | Any positive finding to the “Daily Health Check” sent an automatic email notification to the care coordinator and treating surgeon, who could then contact the patient to address the finding. Patients also had access to a chat feature which sent direct messages to their health care provider team. Patient questions were either answered via the chat function or a telephone call, at the discretion of the treating physician. | There were 63 (55%) patients that used the app’s messaging feature, for a total of 871 messages (including patient and treating team responses) over the study period. A total of five patients used the photo function for wound concerns, and two required intervention in the clinic. |
| Felbaum (2018) [53] | TrackMyRecovery | An app for the perioperative care of surgical patients could serve as a patient engagement tool, improve patient–physician communication, and provide a solution to the episodic and disconnected care of patients, as what happens between health care visits and after discharge is important and strongly predictive of outcomes. We hypothesized that the app should improve compliance with instructions, prevent canceled surgeries, reduce complications and readmissions, and improve patient satisfaction. | Patients received specific, timely, push notification (text message) reminders before and after their surgeries. Patients could send pain scores and wound images through the app securely to the web portal. Providers received automatic email notifications of these submissions and could send messages back to the patient’s app to enhance communication. | Primary care physician | Mobile app | Community/Home/Retirement home | 30 days post discharge | Not reported | Providers received automatic email notifications of these submissions and could send messages back to the patient’s app to enhance communication. | Fifty-four of the 56 patients successfully registered, downloaded, and used the app and read and complied with instructions both before and after surgery. There were no cancelled surgeries. |
| Ganapathy (2017) [54] | Patient Buddy | A proportion of readmissions in cirrhosis, especially because of hepatic encephalopathy could be avoided through patient and caregiver engagement. We hypothesized that encouraging communication between patients, caregivers and the clinical team and increasing education through an innovative App (Patient Buddy) which encourages early intervention would be feasible in patients and caregivers with cirrhosis in a proof-of-concept study. | Cirrhotic inpatients with caregivers were enrolled on separately assigned devices loaded with Patient Buddy, where they were trained on entering medication adherence, daily sodium intake and weights, and weekly cognitive (EncephalApp_Stroop) and fall-risk assessment and were educated regarding cirrhosis-related symptoms. These were monitored daily through a Patient Buddy loaded iPad by the clinical team. The App sent automatic alerts between patient/caregivers and clinical team regarding adherence and critical values. | research team/ outpatient clinics if needed | Mobile app | Community/Home/Retirement home | 30 days post discharge | Once daily | Over the course of the study, alerts were monitored by the team using a central iPad with Patient Buddy to provide care as needed. If there were critical values or missing entries of critical medications, daily weights or sodium intake over 24 hours, automatic alerts were generated for the research team and to the patients and caregivers. | Of the forty patient-caregiver dyads, four did not complete App entries (one did not enter anything at all, three stopped entering at days 12, 15 and 21 post-enrolment) while two were unable to achieve adequate connectivity at home. Most patients and caregivers (n = 24) were not able to adhere to daily sodium entries (total of 734 alerts due to missed sodium intake) consistently because they thought them to be too cumbersome. |
| Gollish (2019) [55] | myHip&Knee | To improve patient education, engagement and self-management through interaction modules accessible on mobile devices. | A key feature of myHip&Knee is the daily health check that begins after discharge from the hospital. Patients answer a series of questions related to symptoms, pain, range of motion and activity, and then, based on their responses, recommendations are generated. There is a library of information, which is provided in a variety of formats (videos, images, text), to encourage patient engagement and enhance patient education. Some of the key topics covered include pain management, wound healing, rehabilitation and common postoperative concerns such as constipation. | app does not communicate with healthcare team, but indicates who to contact and when. | Mobile app | Community/Home/Retirement home | 28 days post discharge | Once daily | There are differences in the daily health check questions depending on if the patient had hip or knee replacement surgery. Based on responses to questionnaire, recommendations are generated. | Not reported |
| Gunter (2018) [56] | WoundCheck | Surgical site infections (SSI) are the most common hospital-acquired infections among surgical patients and the leading cause of hospital readmission after surgery. The use of a smartphone app could help in identifying these complications can allow surgical care providers to visually inspect wounds from a distance and therefore enable outpatient management instead of hospital readmission. | There are 2 phases of the app: an image- taking phase in which participants take up to 4 digital images of their surgical wound, and a brief survey of yes or no questions about recovery, with particular attention paid to the surgical wound. Survey questions were developed to capture information not as easily appreciated from images, such as drainage and odor. | Nurse practitioner, Primary care physician | Mobile app | Community/Home/Retirement home | 14 days post discharge | Once daily | Daily a clinician on the inpatient vascular surgery service reviewed submitted images and survey responses, if concerns detected, they called participant for additional information and made recommendations for additional care as indicated. | Forty-five percent of participants (18 of 40) submitted data every day for the full 2 weeks. Those that did not missed an average of 1.4 days, giving an overall daily submission rate of 90.2%. |
| Habib (2021) [57] | Medication adherence mobile application | To assess the feasibility of a larger evaluation of Smart About Meds (SAM), a patient-centered medication management mobile application. To evaluate SAM’s potential to improve outcomes of interest, including adherence to medication changes made at hospital discharge and the occurrence of adverse events. | Application included  Images of the purchased pill and information displayed in the patient's medication list; information on the indications for treatment, harms, and benefits of medications; adherence alerts; side effect checker; drug-drug interaction checker; caregiver connect; rate my med; pharmacist connect (secured messaging service); and pharmacist-facing dashboard | Pharmacist | Mobile app | Community/Home/Retirement home | 30 days post discharge | Not reported | Medication information based on each individual's need | Utilization rates were calculated as median number of times each feature was accessed by patients and caregivers |
| Hagglund (2015) [58] | Home Intervention System (OPTILOGG) | The overall purpose of education and other intervention modalities, for instance home visits, nurse-led clinics and telephone support is to improve self-care and patients adherence. Some interventions in particular have demonstrated promising results that clearly state the importance of education about the disease, and how education not only empowers the patient and provides a sense of being in control but also has an impact on health-related quality of life. | The tablet contained information about heart failure and lifestyle advice according to current guidelines. it also showed present dose of diuretic, changes in patient-measured weight and health-related quality of life over time. | Nurse, Primary care physician, non-healthcare professional installed system into patients home | Web-based application, specialised software, a tablet computer (tablet) wirelessly connected to a weight scale | Community/Home/Retirement home | 90 days post discharge | Once daily | Every fifth day a Visual analogue Scale turned up where the patient could evaluate his/her perceived health status. | The adherence to using the system was high with a median adherence of 88% [iQr: 78%, 96%]. |
| Heiney (2020) [59] | Healthy Heart | Recent studies suggests that use of mHealth apps is associated with reductions in mortality and hospitalizations, along with improved adherence to therapy and enhanced quality of life. African Americans have higher mortality rates from heart failure than other racial groups in the United States. Therefore, self-management of heart failure may improve health outcomes for African American patients. | The Healthy Heart app incorporates the following evidence-based features to promote self-management: one-way messages, journaling (i.e., weight and symptoms), graphical display of data, and customized feedback (i.e., clinical decision support) based on daily or weekly weight. The educational messages included information on diet, sleep, stress, and medication adherence. | Nurse, Primary care physician | Mobile app, journalling and customized feedback | Community/Home/Retirement home | 30 days post discharge | A total of 3 messages were sent daily: (1) a reminder to weigh every morning, (2) an educational message, and (3) motivational messages. | The final component was customized feedback (i.e., clinical decision support) that sent an alert message if the weight exceeded standards set by the home health protocol for monitoring heart failure. | One participant was lost to follow up at the second assessment. Thus, 11/12 (92%) participants completed the study. |
| Heuser (2019) [60] | SeamlessMD | Adherence to behavioral changes such as exercise and dietary restrictions has been shown to improve long- term weight-loss outcomes. Thus, patient education, engagement, and adherence to prescribed protocol are key components of care in bariatric patients. Mobile technology may improve surgical outcomes by promoting patient adherence to perioperative protocols and enabling early identification of postoperative complications. | The app provided pre-surgical and post-surgical educational information and helped patients make decisions on how to assess and follow up on symptoms. In addition, patients were asked to answer questions about their symptoms in the Daily Health Check survey for 30 post- operative days. The questions asked patients about their postoperative symptoms in addition to self-care and adherence to their recovery program as well as various symptoms and health care utilization. | "clinical staff"/ research team | Mobile app | Community/Home/Retirement home | 30 days post discharge | Once daily | Depending on the survey response, patients were advised if they were on-track or need to seek symptom management by reviewing self-care education through the resource library feature, by calling the clinical staff, or by visiting the emergency department. | 66.2% of patients completed the daily health check survey at least once in the first week after the surgery. This number decreased with time, with 53.6%, 46.9%, and 38.2% of patients completing the survey at least once a week in weeks 2, 3, and 4, respectively. |
| Heyworth (2014) [61] | Secure Messaging for Medication Reconciliation Tool’ (SMMRT) | To improve medication safety among patients recently discharged from hospital using a secure messaging for medication reconciliation tool. | Display of patient’s list of medications (generic name, dosage strength, formulation (e.g., tablet or inhaler))  Directions for medication administration (e.g., take one-half tablet each morning and each evening).  Interactive component to perform medication reconciliation independently at home by checking ‘yes’ or ‘no’ to each listed medication and add free-text questions or comments. | Pharmacist, Nurse, Primary care physician | Web-based application | Community/Home/Retirement home | Not reported | Not reported | Not reported | Not reported |
| Highland (2019) [62] | mCare system | Typically, post-surgical follow-up calls enable nurses to assess a patient’s condition, provide tailored education, and improve the patient’s experience. We hypothesized that an mHealth app might reduce hospital staff burden completing follow-up with patients and be a more acceptable assessment method, per patient report. | The website portal was used to push text message reminders to complete the postsurgical questions. | Nurse | Mobile app | Community/Home/Retirement home | 10 days post discharge | Once on day 2 and once on day 7 | On day 2, participants were asked to report whether the effects of the block had worn off (no, yes). If the effects had not worn off, they were prompted again on day 7 to indicate if and when the block effects had worn off. | Participants (N = 50) were randomized to the mCare group (N = 24) or the SOC (N 1⁄4 26) group. One mCare participant and four standard-of-care participants were withdrawn due to surgery cancelations or not receiving a regional anesthesia block. Therefore, five participants were withdrawn and not included in the analyses |
| Holzer (2022) [63] | HealthFlo | We developed a novel VTE Transitions of Care Bundle using a patient-facing mHealth application and a dedicated patient navigator. Secondary aims were to reduce rates of recurrent VTE, bleeding complications, and hospital readmissions. | The app features secure messaging, patient education materials, and daily medication reminders. | Social worker, | Mobile app | Community/Home/Retirement home, Rehabilitation, Long-term care/ Nursing home (24hr care) | 30 days post discharge | Once daily | Not reported | Application uptake was noted for 32 of 89 (36%) patients in the intervention group. |
| Houchen-Wolloff (2021) [64] | Web-based Self-management Program of Activity Coping and Education | To assess the feasibility and acceptability of a web-based self-management program. | Comprehensive package of exercise (Record of aerobic walking exercise, symptom diary); Self-management education (short-term goals, knowledge tests on COPD and exercising safely, reading or watching videos inhaler techniques or healthy eating) Enhance communication (moderated blog section for patients to share experiences with others, and connect with an expert facility) | Nurse | Web-based application | Community/Home/Retirement home | 365 days post discharge | Not reported | Not reported | Not reported |
| Ilaslan (2022) [65] | Web-based application for training and telephone follow-up for patients with heart failure | To develop and test the feasibility of a novel web-based application called MyPath to Home that can be used to manage the personalized needs of geriatric rehabilitation patients during their transition from the hospital to home. | Information about heart failure; Information on control of symptoms; Recommendations for lifestyle changes, health behaviors, and practices; Written texts, visuals in picture format, shapes, and photographs;  Body mass index and a calorie-calculating engine; User and administrator interface pages. | Nurse | Web-based application | Community/Home/Retirement home | 90 days post discharge | Four-session telephone follow-up, and one text message weekly | Not reported | Adherence not reported. 6 participants lost to follow-up |
| Indraratna (2022) [66] | TeleClinical Care | To investigate the feasibility, efficacy, and cost-effectiveness of a smartphone app–based model of care (TeleClinical Care [TCC]) in patients discharged after ACS or HF admission | Control group: Usual care Intervention group: Usual care and TeleClinical Care (TCC) which involved checking blood pressure, pulse rate, and weight daily with a sphygmomanometer. | Primary care physician, Cardiac nurse practitioner, Cardiologist | Mobile app, Wearable device | Community/Home/Retirement home | 30 days post discharge | Recommended daily use. 3 weekly educational push notifications to promote healthy behavior choices, including dietary advice, physical exercise, and smoking cessation | Not reported | Adherence to intervention not reported. 164 randomized, 156 analyzed due to loss to follow-up |
| Johnson (2022) [67] | HF-SMART | To determine whether a mobile health program can assist patients in the self-management of heart failure and foster lifestyle behavior modifications that promote and improved quality of life. | Educational content tailored to patients with chronic heart failure (videos and daily prompts) Alerts that directed patients to contact medical personnel in the event of urgent health issues Active monitoring of patient HF data by nurses Interactive feedback of patients’ symptom assessment with biometric tracking Reminders for medication adherence. | Nurse, Research assistant | Web-based application | Community/Home/Retirement home | 30 days post discharge | Daily | Interactive feedback of patients’ symptom | 14 of 16 (87.5%) participants completed at least 30 days of the program |
| Kang (2022) [68] | Web based discharge education programme | To assess the feasibility of implementing a web-based discharge education programme for general surgery patients both prior to and after hospital discharge | Three components: (1) post general surgery warning signs; (2) post general surgery everyday care instructions; and (3) a video on surgical wound care and signs of wound complications.  Traffic light system; A tiered system advised patients on how to act based on whether a symptom was normal or abnormal following general surgery. | Pharmacist, Dietitian, Nurse, Surgeon | Web-based application | Community/Home/Retirement home | 30 days post discharge | Recommended regular use | Not reported | Twenty-three (27%) lost to follow-up. Google Analytics was used to monitor patients' compliance with the intervention |
| Kargar (2020) [69] | Self-care educational mobile application for burns | To assess the effect of a self-care educational mobile application on the QOL of victim patients with hand burns. | Control group: Only received the ordinary training presented in the burn center such as educational pamphlets and in-person training provided by nurses. Intervention group: Self-care training and application. | Physiotherapy, Dietitian, Nurse, Surgeon | Mobile app, Web-based application | Community/Home/Retirement home | 60 days post discharge | Daily use recommended | Not reported | Not reported |
| Keng (2020) [70] | Home to Stay digital Program after colorectal surgery | To perform an initial assessment of patient uptake, outcomes, and satisfaction with the Home to Stay Program and use this information to evaluate if the Home to Stay system should be fully adopted into clinical practice. | Customized to the needs of patients who have undergone colorectal surgery including  (1) a “Daily Health Check,” for the patients to report on their postoperative recovery;  (2) picture taking capability for patients to photograph their incisions and stoma to share with their health-care providers; and  (3) educational information on postoperative care and self-management at home | Physician assistant | Mobile app | Community/Home/Retirement home | 30 days post discharge | Daily use of app | App qualities customized to the needs of patients who have undergone colorectal surgery | Ninety-three patients ultimately logged into and used the app at least once, for an overall participation rate of 88% (93/106). |
| Kersey (2022) [71] | Mobile Health Platform For Strategy Training In Inpatient Stroke Rehabilitation (iADAPT) | To examine whether strategy training using a mobile health platform (iADAPT) is feasible during inpatient stroke rehabilitation and following discharge | Participants using mobile app: Received strategy training protocol via mobile health platform which involved 1) Identifying meaningful activity-based goals using the Activity Card Sort. 2) Participants introduced to the global strategy (Goal–Plan–Do–Check) and attempted to apply the global strategy to their first activity-based goal.  3) Participants asked to identify strategies they learned that they could apply to other activities | Not reported | Mobile app | Community/Home/Retirement home | Not reported | Both groups received 7 in-person sessions during inpatient rehabilitation and 3 remote sessions following discharge | Not reported | Patient adherence indicated by the number of goal trials attempted. Attrition reported in the form of participants lost to follow up, those that withdrew, and other reasons |
| Khan (2018) [72] | Activeheart portal | To determine how cardiac patients use the Activeheart.dk digital toolbox | Portal provided information about post-operative precautions and exercises, lifestyle changes, psychological issues and coping strategies. | Not reported | Web-based application | Community/Home/Retirement home | 30 days post discharge | Not reported | Not reported | Not reported |
| Khanwalkar (2019) [73] | Digital patient engagement (DPE) platform | Patient-reported outcome measures (PROMs) have become essential tools to assess patient satisfaction, functional outcomes, and the quality of care delivered in the healthcare system. A novel, automated, Internet-based digital patient engagement (DPE) platform has been developed to maximize patient interaction with physicians, allowing higher response rates without the need for tedious and time-consuming patient follow-up. | The mobile phone application relayed patient-reported outcome measurements (PROMs), including pain (assessed on a 0 to 10 visual analogue scale [VAS] every other day for 2 weeks, as well as the patient-reported outcome measure information system [PROMIS] pain interference short form 4a at baseline, 2 weeks, and 3 months) and timing of return to work. | rhinologists in outpatient clinic | Mobile app | Community/Home/Retirement home | 14 days post discharge | Every other day | Not reported | Of 288 patients enrolled into the DPE mobile platform in the rhinology clinic, 249 (86.5%) engaged with the application to provide some degree of clinical data and 223 (77.4% response rate) provided clinical data relevant to our planned analyses. |
| Kim (2016) [74] | iGetBetter (iGB) program | Patients undergoing total knee arthroplasty can significantly benefit from the use of app-assisted medical support because it is patients' compliance and actions during the preparatory and recovery processes of the procedure that play a crucial role in the final outcome. | Patients logged whether they had completed their respective care plan activities for the day or not; they also responded to reminders communicated through the app. These reminders are based on common preoperative and postoperative protocols that are implemented in our pathway for patients undergoing total knee arthroplasty. Data from these manual logs are received in real-time by the clinical team through a web portal. | Primary care physician | Mobile app | Community/Home/Retirement home | 30 days post discharge | Once daily | All patient answers could be monitored by the clinicians via an iGB clinician dashboard, and the system was equipped to trigger alerts for the clinician if patients provided answers that fell outside ranges the clinician deemed acceptable. | With regard to the postoperative phase, users logged in on an average of 17.77 out of the total of 30 days (59.2% of days, range of 4-30 days). |
| Knapp (2021) [75] | PeerWell | To allow for patient education on understanding of the disease, its treatment options, and best practices for postoperative care. | The App provides comprehensive patient education using a series of modules delivered at set intervals preoperatively and postoperatively. | Not reported | Mobile app, Web-based application, Text messaging (SMS) | Community/Home/Retirement home | 84 days post discharge | Not reported | The application is designed to present the information to the patient at set intervals based on the date of surgery. | As time after surgery increased, patient engagement gradually decreased, dropping from 67% at 2 weeks, to 30% at 12 weeks |
| Kooij (2021) [76] | Self-Management App for High-Risk Patients with Chronic Obstructive Pulmonary Disease | To assess the effectiveness of a self-management app for high-risk COPD patients | Lung Attack Action Plan (Guide patients in recognizing and responding to changes in their symptoms), Personalized medication overview, Information and education (COPD condition, nutrition, physical activity, and smoking cessation), Weekly questionnaires and monitoring (Weekly Clinical COPD Questionnaire (CCQ) and the Hospital Anxiety and Depression Scale (HADS)), and  Video consultations | Nurse, Nurse practitioner, Pulmonologist | Mobile app | Community/Home/Retirement home | 56 days post discharge | Weekly sessions over a period of 8 weeks | Medication information tailored per participant | Adherence to treatment (P=.14), or in relation with app use (P=. 92); 2 patients lost to follow-up (died) |
| Kristjánsdóttir (2013) [77] | Smartphone-Based Intervention for Chronic Widespread Pain | To study the efficacy of a 4-week smartphone-delivered intervention with written diaries and therapist feedback following an inpatient chronic pain rehabilitation program | Control group: Pain mechanisms and CBT-based pain management (approximately 20 hours), motivational interviewing sessions (4 hours), aerobic exercise (outdoors, in the pool, and at the gym), stretching, and relaxation sessions. Intervention group: Face-to-face session, web-based diaries, written situational feedback, audio files with guided mindfulness exercises | Nurse | Mobile app, Web-based application | Community/Home/Retirement home | 30 days post discharge | 1 face-to-face session and 4 weeks of written communication via a smartphone | Therapists contacts with patients tailored and patient diaries subjective | Adherence not reported. Twenty-one withdrew from the intervention group (30.4%) and 2 withdrew from the control group (3.0%) |
| Kummerow Broman (2015) [78] | My Health at Vanderbilt (MHAV) | Many patients seek greater accessibility to healthcare. Meanwhile surgeons face increasing time constraints due to workforce shortages and elevated performance demands. Online postoperative care may improve patient access while increasing surgeon efficiency. | Vanderbilt’s online patient portal (MHAV), provides a way for patients to access health data and communicate with their care providers in a secure setting outside of traditional clinic visits. On the date of the online visit, the patient would submit a survey that included symptom-based questions, image upload links, and free text boxes for participant comments. | Surgeon | Web-based application | Community/Home/Retirement home | 28 days post discharge | Postoperative clinic visits were scheduled for about four weeks after the planned operation. Online visits were scheduled for one week prior to the clinic visit. | Surgeons responded to patients about their symptom reports and wound images in an asynchronous fashion using the online portal, which were automatically documented in the electronic medical record. | Among 70 participants to whom the online visit link was sent, 51 completed an online postoperative visit (73%). Excluding participants who did not complete clinic or online visits (4) and those who were initially randomized to the clinic-then-online visit sequence (6), 84% (51/61) of eligible patients completed the online visit. |
| Layton (2014) [79] | Smartphone Based Application to Monitor Outpatient Discharge Instruction in Compliance Cardiac Disease Patients | To investigate the feasibility and acceptability of a smartphone iOS application to monitor and assist with patient medication compliance, education, home exercise, symptom changes, and transition to outpatient care team after hospitalization | Daily educational material, medication reminders, doctor appointment reminders and monitored activity level. Process measurements, such as user engagement, daily task completion, and perceived value of the application to the patient, recorded. Activity level, medication compliance, follow-up care, enrollment into support programs. | Nurse, Primary care physician | Mobile app | Community/Home/Retirement home | 60 days post discharge | Daily monitoring of medication compliance, physical activity, follow-up care, symptoms, and reading of education material | Elements of care and follow-up based on individual's needs | Over the course of the study, only 19% of patients used the application during week 7, indicating poor adherence. For attrition, A total of seven patients completed 1–30 days and four completed >31 days. |
| Lee (2022) [80] | Mobile app follow-up for same day discharge | To investigate the feasibility of SDD protocol with postdischarge follow-up using a mobile phone app in patients undergoing elective minimally-invasive colectomy | Daily health check questionnaire on GI recovery, fever, and pain. Mobile app chat feature that sends direct messages to health care providers. | Surgeon, Anesthesiologist | Mobile app | Community/Home/Retirement home | 7 days post discharge | Messages with provider monitored from 7 AM to 5 PM, 7 days a week | Not reported | Not reported |
| Lee (2022) [81] | mHealth remote post discharge monitoring | To compare outcomes after SDD for minimally invasive colectomy using mHealth or telephone remote post-discharge follow-up. | Daily health check questionnaires were administered upon discharge and continued to POD 7, focusing on GI recovery, fever, and pain.  Chat feature through the mobile app which sent direct messages to their health care provider team. | Surgeon, Anesthesiologist | Mobile app | Community/Home/Retirement home | 7 days post discharge | Patients were advised that chat Messages with provider monitored from 7 AM to 5 PM, 7 days a week | Not reported | Not reported |
| Liu (2021) [82] | Together | The ultimate goal was to improve quality of life and self-efficacy, which can provide a comprehensive picture of the individual's physical-psychological health and social domains. | The study group received five follow-ups conducted by trained nurses through the app, which had four core functions, namely remote assessment, health education, interdisciplinary referral, and patient interaction, at weeks 2, 4, 6, 8, and 12 following discharge. | Physiotherapy, Occupational therapist, Nurse, Primary care physician | Mobile app | Community/Home/Retirement home | 84 days post discharge | at weeks 2, 4, 6, 8, and 12 following discharge | The nurse could remotely evaluate patients’ functioning by questioning patients via telephone according to preset instructions on the app. | The overall effective follow-up rate in this study was 96.1% (98/102), and the attrition rate was 3.9% (4/102). |
| Li (2022) [83] | Digital therapeutics (DTx) | Recent advances in digital therapeutics (DTx), which delivers medical interventions directly to patients using evidence-based, clinically evaluated, technology-based software algorithms or apps to facilitate disease management, such as smartphones and technology, have made DTx a promising solution for secondary prevention management of chronic diseases. | The DTx system includes a physician portal, a health manager portal, and a patient portal and contains 3 modules (a discharge module, home management module and follow-up module). The intervention group had their blood pressure and heart rate data transferred through Bluetooth connection to the app, or they could also input the data on the app by themselves. In addition, an automatic alarm was set up in the patient portal to help manage their daily medical regiment. Participants in the intervention group could also record symptoms and notes in the DTx app. All data recorded by the home management module are shared with trial staff physicians and nurses in the physician portal. | Nurse, Primary care physician | Mobile app | Community/Home/Retirement home | 365 days post discharge | Once daily | Patient education materials and instructions on medication were also provided in the app according to the diagnosis at discharge. | Randomization yielded 49.3% (148/300) and 50.7% (152/300) patients in the intervention and control groups, respectively. However, 2% (6/300) of the patients withdrew consent after randomization, and 1.3% (4/300) of the patients were lost to follow-up during the study. Of the 300 patients, 290 (96.7%) patients were followed up for 12 months after randomization. |
| Lou (2022) [84] | Mobile health supported volunteer-assisted self-help (mVS) intervention to enhance spiritual well-being | To enhance spiritual well-being for discharged older patients through a medical social-academia tripartite collaboration using a theory-driven mobile health supported volunteer-assisted self-help (mVS) intervention. | Educational sessions on varying topics:  Session1 (meaning of life and achieving optimal being) Session 2 (Spiritual breathing) Session 3 (Self-care in living a meaningful life) Session 4 (role of family support) Session 5 (Friendship as support system) Session 6 (Forgiveness) Session 7 (Sources of life energy) Session 8 (Reviewing meaning of life & prepared for separation anxiety) | Social worker | Mobile app | Community/Home/Retirement home | Not reported | 8 session mVS intervention that conducted on an individual basis | Not reported | Adherence not reported |
| Lyu (2021) [85] | Nurse led web-based transitional care program | To develop a web-based transitional care program and evaluate its effects on the glycemic control and quality of life of Chinese patients with type 2 diabetes and to explore the mediating roles of self-efficacy and treatment adherence. | Program collected data including demographic information, HbA1c levels measured based on vein blood by nurse; Chronic Disease Self-Efficacy Scale; Medical Outcomes Study 36-item Short-Form Health Survey; and Treatment Adherence Scale for Patients with Diabetes. | Nurse | Web-based application | Community/Home/Retirement home | 90 days post discharge | Daily use recommended | Not reported | Self-designed treatment compliance scale available to assess adherence. 10 participants lost to follow-up (106/116) |
| MariaGomez (2022) [86] | mHealth application in patients with type 2 diabetes | Not reported | Intervention group: ClouDi website to automatically upload glucose measurements. Measurements reviewed weekly and recommendations for insulin adjustments sent via text message if necessary . Assessed by nutrition service  Control group: Assessed by nutrition service, received education in the management of diabetes medications an diet. | Endocrinologist | Mobile app, Web-based application | Community/Home/Retirement home | 90 days post discharge | Daily use of website recommended | Information collected and feedback based on patient | Not reported |
| Marvel (2021) [87] | Acute myocardial infarction digital health intervention | To determine if patients with AMI using DHIs have lower 30-day unplanned all-cause readmissions than a historical control | Manage medications (track daily adherence, indication, and side effects) Monitor their vital signs (heart rate, BP, weight, mood, and steps) Learn about the risk factors for CVD and lifestyle modification through educational articles  Animated videos (Nucleus Media) Schedule and track follow-up appointments Connect with their care team Store health information such as stent and insurance cards. | Nurse, Primary care physician, Cardiologist | Mobile app, Wearable device | Community/Home/Retirement home | 30 days post discharge | Not reported | Not reported | Corrie app able to track daily patient medication adherence. |
| Metilda (2021) [88] | Aimeo | Smart phone–based applications (Apps) for home-based care can help in improving the compliance to discharge instructions, as well as a monitored medical care, for the patients in remote areas. The management of neurological patients aims to reduce their disability and adapt themselves within society. | Patient’s or caregivers update the vitals, adherence to discharge advice the App which is shared with the nurse and treating doctor, so that remote patient management is possible. Patients can upload their queries as JPG, PDF, videos, or chat in the mobile App. The call is sent to the patient from call center regarding the query and if medical clarification needed the call is connected to the nurse or doctor resolves the queries of patient or caregivers, thereby managing the patients remotely. | Nurse, Primary care physician | Mobile app | Community/Home/Retirement home | 60 days post discharge | Once daily | The individualized discharge summary was embedded into the mobile App with educational videos in it. The patient or caregiver was encouraged to update the data in the mobile App as per their discharge advice and reminder SMS were sent to the patients for discharge compliance. | According to the data updated by the patients in the mobile App, 66% patients used 40 to 60% of mobile Apps for first month which was reduced to 8% patients in the second month. Also, 22% patients in the first month and 82% patients in the second month used below 40% of mobile App according to the data updated by them. |
| Park (2019) [89] | Digital health monitoring for heart failure patients | To examine the feasibility of using digital health monitoring in real-world home settings, ascertain patient adoption, and evaluate impact on 30-day readmission rate. | Heart Failure education content, SMS or text reminders, Tracking patient-reported outcomes via preselected symptom checkboxes | Nurse, Cardiologist | Mobile app, Web-based application | Community/Home/Retirement home | Not reported | Recommended daily use | Physicians contact patients based on individual judgment of patient changes | Not reported |
| Paruchuri (2021) [90] | Wellframe | To improve adherence to cardiac rehabilitation after coronary artery disease events, which has been demonstrated to reduce both mortality rates and hospital admissions. | The mobile health platform consisted of a patient- facing mobile app, a clinical dashboard and a suite of clinical programs with configurable rules. The patient mobile app featured a personalized adaptive daily health checklist that included reminders to engage in health behaviors and a series of personalized, interactive surveys, articles and encouragement. | third party non-medical health coaches | Mobile app | Community/Home/Retirement home | 90 days post discharge | Once daily | A personalized adaptive daily health checklist and a series of personalized, interactive surveys, articles and encouragement | All 118 participants utilized the smart- phone-based application.  Engagement rates were durable across 30- and 90-day periods. At 30- and 90-days, 68 (57.6%) and 63 (53.5%) participants respectively opened at least 50% of the articles suggested by the app. |
| Peng (2022) [91] | Mobile continuous nursing platform | The continuous nursing model based on Internet technology can effectively break through the limitations of region, time, and economy, realize the continuation from hospital to family/social care, optimize the health management of patients, enhance the self-management ability of patients, and meet the actual health needs. | Full-time nurses needed to make the content of health education as an electronic web page and uploaded it to the Internet continuous nursing platform every day, which included T-tube placement position and fixation method, observation of bile-related traits, treatment method of T-tube slippage, psychological counselling, diet, and medication guidance. Additionally, online and offline joint guidance for patients and their families was conducted to ensure proper care of the patient. | Nurse | Mobile app, The patient’s family was given a contact method of the nurse on duty in order to cope with emergencies. | Community/Home/Retirement home | 14 days post discharge | Once daily | Online and offline joint guidance for patients and their families was conducted to maintain the dry incision dressing; keep the surrounding skin clean; selection, fixation, and replacement of drainage bag; observation of bile colour, quantity, and character changes; activity method carrying T-tube; drug use methods; and adverse reactions, and timely psychological counselling was carried out. | Not reported |
| Pickens (2019) [92] | SeamlessMD | Patient-reported outcomes (PROs) have been well validated for measuring patient experience during perioperative care and are becoming foundational in the growing movement toward patient-centered care. The use of a mobile application for PRO collection specifically within an ERAS! program has yet to be explored for the often deconditioned and comorbid hepatopancreatobiliary population undergoing major abdominal surgery. | The application provides a patient- and surgeon-specific portal to their individual perioperative surgical plan guided by the established Enhanced Recovery After Surgery (ERAS) pathway. Preoperatively, patients are prompted to access a customized digital educational library reviewing details of their medical condition, scheduled operation, and anticipated Enhanced Recovery After Surgery (ERAS) expectations. Beginning on the day of surgery, the application then provides prompts to complete daily surveys to track symptoms, opiate use, anxiety and quality-of- life scores, and Enhanced Recovery After Surgery (ERAS) pathway compliance. These surveys are prompted daily in hospital and after discharge until 30 days postoperatively. | Nurse, Surgeon | Mobile app | Community/Home/Retirement home | 30 days post discharge | Once daily | Responses to the daily health checks trigger recommendations customized specifically by the hepatopancreatobiliary (HPB) surgeons for education and reassurance, guidance for continued self-care at home, directions to call the HPB office nurse, or instruction to seek immediate medical attention at the nearest emergency department. | Patient adoption was high with 93% (114/122) of enrolled patients using the application at least once before surgery. Immediate postoperative engagement remained high at 88% (107/122). Engagement after discharge showed the highest decline in participation yet still remained above 50% (63/122). |
| Ponder (2020) [93] | Smartphone app with a digital care pathway for patients undergoing spine surgery | To design, develop, and evaluate the acceptability and feasibility of a novel planning-, outcomes-, and analytics-based smartphone app called ManageMySurgery (MMS) in patients undergoing elective spine surgery (MMS-Spine). | Intervention facilitates shared decision-making between patients and caregivers.  Improves patient engagement and workflow. | Surgeon, Psychometrics, and computer scientist. | Mobile app, Web-based application | Community/Home/Retirement home | 30 days post discharge | Not reported | Not reported | Adherence not reported. Nine patients lost to follow-up |
| Pooni (2022) [94] | Post-discharge Home to Stay app mobile app | To evaluate the effect of a postdischarge app on 30-day readmissions and patient reported outcomes following colorectal surgery. | Daily Health Check, Picture taking capability to photograph incisions and stoma, Educational information on post-operative care and self-management at home | Physician assistant, Surgeon | Mobile app, Web-based application | Community/Home/Retirement home | 30 days post discharge | daily use recommended | Daily Health Check participants received tailored recommendations, including relevant education modules, to contact the surgical team or go to an emergency room if needed | Adherence not reported. 29 participants lost to follow-up |
| Pronk (2020) [95] | PainCoach app | To manage pain better and potentially decrease opiate use, an eHealth app named PainCoach was developed. This app aims to help patients control their pain better in the initial period at home after total knee replacement, including optimal use of the available pain medication. | After only entering the date of surgery as patient data, the app allowed patients to input their pain level (no pain, bearable pain, unbearable pain, or untenable pain) whenever they wanted until day 14 after surgery. Based on the patient’s input and taking into account the number of days after surgery, the app provided advice on pain medication use, physiotherapy exercises including videos, use of ice or heat packs, rest, immobilization of the operated leg, and when to call the clinic. | Nurse | Mobile app | Community/Home/Retirement home | 14 days post discharge | Once daily | Based on the patient’s input and taking into account the number of days after surgery, the app provided advice on pain medication use, physiotherapy exercises including videos, use of ice or heat packs, rest, immobilization of the operated leg, and when to call the clinic. | The response rates for the daily questionnaires at home were 91% in the PainCoach group and 89% in the control group. |
| Pugliese (2019) [96] | RecoverNow | Stroke survivors frequently experience a range of post-stroke deficits. Specialized stroke rehabilitation improves recovery, especially if it is started early post-stroke. However, resource limitations often preclude early rehabilitation. Mobile technologies may provide a platform for stroke survivors to begin recovery when they might not be able to otherwise. | RecoverNow is a comprehensive rehabilitation platform including cognitive and fine-motor therapy (often the therapeutic domain of occupational therapists) in addition to speech language therapy. The platform was designed to include a therapist-only web-based administration portal with tablet usage monitoring features that allowed therapists to track patient therapy engagement and to remotely modify therapy content. | Occupational therapist, Speech therapist | Mobile app | Community/Home/Retirement home | 90 days post discharge | Once daily | A speech language pathologist or occupational therapist prescribed participants preselected, modality-specific therapeutic apps for stroke-induced deficits related to communication, cognition, and fine-motor ability based on individualized standard of care assessments. | Participants were non-adherent to tablet-based therapy inside and outside of acute care, using RecoverNow for a median of 12 minutes a day. Retention was high with 23 of 30 patients participating in follow-up interviews (77% retention rate) and all but 3 of the 23 interviews (87%) were successfully completed. |
| Reid (2012) [97] | CardioFit internet-based expert system | To compare CardioFit to usual care to assess its effects on physical activity following hospitalization for acute coronary syndromes. | Control group: The usual care group received physical activity guidance from their attending cardiologist and an education booklet. Intervention: Received a personally tailored physical-activity plan upon discharge from the hospital and access to a secure website for activity planning and tracking | Cardiologists, exercise specialists, and behavioural scientists | Web-based application | Community/Home/Retirement home | 183 days post discharge | Each participant logs their daily activity and completes five online tutorials (at weeks 2, 4, 8, 14, and 20). Physical activity plans were developed after each tutorial. | Those in the intervention group received personally tailored physical-activity plan upon discharge from the hospital and access to a secure website for activity planning and tracking | CardioFit participants completed an average of 2.7 of the maximum five tutorials, and 61.7% completed at least three of them. Participants who were lost to follow-up reported |
| Requena (2019) [98] | Eir (Eir Solutions AS, Norway) | Tools for active communication with the patients after early discharge from the hospital will be important to avoid or address post-operative complications. | The patient module was designed for patient-reported postoperative symptom assessment and medication registration after fast-track knee arthroplasty. It consisted of measurements of pain and side effects and detailed registration on use of analgesic drugs. | Nurse, Primary care physician | Web-based application | Community/Home/Retirement home | 8 days post discharge | Once Daily | Not reported | 100% of the patients (134/134) completed the randomized control trial. |
| Rian (2022) [99] | FARMALARM | Telemedicine has already shown an improvement in the control of specific vascular risk factors like diabetes mellitus or hypertension. We aim to validate the use of FARMALARM, an application (app) for smartphones, in vascular risk factor control as a tool for secondary prevention of stroke. | Farmalarm is an application (app) for smartphones designed to increase stroke awareness by medication alerts and compliance control, chat communication with medical staff, didactic video files, exercise monitoring. | Occupational therapist, Primary care physician | Mobile app | Community/Home/Retirement home | 30 days post discharge | Once daily | On the day of discharge, the occupational therapist uploaded and scheduled on the web-based platform a personalized program for the following 3 weeks, according to the patient diagnosis, risk factors, and prescribed secondary prevention treatment. | Not reported |
| Rosner (2018) [100] | Internet-based Orthopedic Patients' Self-Reports of Postdischarge Complications | Internet-based Orthopedic Patients' Self-Reports of Post discharge Complications | Automated check-in notifications were sent to patients which led the patient to a HIPAA-compliant environment that provided timely materials for that day, including reminders, checklists, educational materials, structured symptom assessments, and patient reported outcome measures. | Primary care physician | Mobile app | Community/Home/Retirement home | 90 days post discharge | Not reported | Patients received automated email notifications based on procedure-specific care plans | Adherence not reported. 86 participants dropped out of study |
| Saunders (2021) [101] | My Hip Journey | To further enhance the postoperative educational experience, patient engagement, self-care, and outcomes across the surgical pathway. | Daily ("My Program") window displays a list of videos and information as well as exercises that had been allocated for them to view or complete that day.  Participants could also communicate with the health care team at the hospital using the communication log within the program. | Health care team (not specified) | Web-based application, email reminders | Community/Home/Retirement home | 42 days post discharge | Once daily | Participants could also invite other health care professionals or support persons to be part of the program. | At the end of phase 4 (6-month follow-up), 66% (33/50) of participants remained in the intervention group and 82.9% (39/49) in the control group. |
| Schenkel (2020) [102] | ActiCare | In the past few years, remote monitoring applications that measure patient pulmonary function and other clinical parameters have emerged as potentially useful tools for improving lung transplant recipient compliance with care and reducing post-transplant complications. | The system leveraged Bluetooth technology to transmit patient vital sign measurements and respiratory parameters to transplant coordinators; allowed face-to-face communication between patients and providers, included educational library complete with customized video content, tutorials, and self-assessments, and relied on humorous memes, inspirational messages, and incentive badges to encourage patient compliance with daily reporting. | Primary care physician, transplant coordination team | Bluetooth-enabled devices and Microsoft Surface tablets | Community/Home/Retirement home | 730 days post discharge | Once daily | The platform allowed for face-to-face communication between patients and providers, included a comprehensive educational library complete with customized video content, tutorials, and self-assessments, | All patients enrolled in monitoring complied with the program, reporting data at least weekly during follow-up, and 53.5% of monitored patients reported data 3 times per week or more. |
| Scheper (2019) [103] | Woundcare | We hypothesized that using a mobile woundcare app with an integrated alert system may lead to increased patient involvement, early detection of wound problems and prevention of chronic prosthetic joint infection. | The app consisted of an introductory page collecting basic patient characteristics followed by daily short questionnaires regarding the patient’s wound. Patients recorded redness, pain (by visual analogue score, VAS), wound leakage, fever and a picture of the wound could be taken. Based on the daily questionnaires, an algorithm created daily a risk-score. | Surgeon | Mobile app | Community/Home/Retirement home | 30 days post discharge | Once daily | Based on a daily questionnaire, an algorithm created daily a risk-score (for infection). If the score exceeded this threshold, an alert message on the smartphone advised patients to contact their treating physician within 24 h. | Forty-one patients (59.4%) used the app until day 30. Nine patients (13.0%) stopped using the app immediately after the first or the second day of use. |
| Schneider (2017) [104] | Technology improve coping for patients after stroke | To examine differences in discharge readiness and postdischarge coping in patients admitted for stroke after the use of individualized postdischarge information/education provided via a technology package compared with current standard discharge teaching methods. | Scripted messages based on core stroke measures were sent by secure e-mail messaging, ensuring that discharge teaching was reinforced. Day 2 message included stroke symptoms and calling 911 if they occur. A message was sent on day 6 discussing stroke risk factors. On day 10, the patient was given information about emotional changes/feelings that may arise after a stroke and what to do about them. | Not reported | Mobile app | Community/Home/Retirement home | 30 days post discharge | Not reported | Messages sent on risk factors tailored to the patient | Adherence not reported. 100 participants enrolled, 86 completed data sets |
| Schubart (2012) [105] | E-Learning Program to Prevent Pressure Ulcers in Adults with Spinal Cord Injury | To provide access to readily available, understandable, and effective Pressure ulcer prevention strategies and practices to patients with spinal cord injury | Skin checks, posture, stretching, mobility (transfers), mobility (wheelchair skills), equipment (cushion), Empowerment to take charge of their own skin care regimen, Awareness of the importance of ongoing equipment maintenance and replacement, Strategies for avoiding common accidents that injure skin | Physiotherapy, Occupational therapist, Nurse, Primary care physician | Web-based application | Community/Home/Retirement home | 14 days post discharge | Designed to be used in multiple sessions | Not reported | One participant withdrew from the study after using the program but did not complete the post-intervention assessment. Adherence not reported |
| Scott (2017) [106] | Postoperative mHealth App | To gauge interest in smartphone app use among patients after colorectal surgery and to better understand factors affecting patient app use in a postoperative, at-home setting. | Daily postoperative self-reporting.  Symptom tracker.  Onscreen reminder for patients to complete the questions by a set time. | Not reported | Mobile app | Community/Home/Retirement home | 14 days post discharge | Not reported | Not reported | 3 patients lost to follow-up |
| Siegel (2016) [107] | Personal health assistant (PHA) stroke app | To increase poststroke patient care and prevent hospital readmission | Manage medication compliance, follow-up instructions, navigate health insurance issues, schedule appointments, and answer patients' questions | Nurse, Nurse practitioner, Physician assistant | Mobile app | Not reported | 30 days post discharge | 24 h a day, 7 days a week | Not reported | Adherence not reported. Three patients enrolled, only two used the app |
| Stapler (2022) [108] | St. Joes Health App | Designed a readmission reduction bundle (phone/tablet patient communication application, pharmacist-led pain management plan, and uniquely staffed post-discharge clinic) that allows earlier intervention on evolving complications and other patient care issues in the outpatient setting. | Patients answer questions related to numeric pain scores and pain management options, anti- coagulants, stoma appliance, sleeping posture, inputs and outputs, and wound appearance and wound care, and they are provided with physical therapy and nutrition recommendations.  The app is monitored 24/7 by the ERP nurse navigator during the day and the on-call physician at night and on weekends. | Nurse, Surgeon | Mobile app | Community/Home/Retirement home | 3 days post discharge | Once Daily | Patient responses are based on green (expected), yellow (standard response from app), and red light (phone call with provider) indicators. This phone call may result in a same-day office visit to investigate the concern. | Not reported.  1052 patients in preintervention group; 668 patients in postintervention group. However not explained why. |
| Su (2021) [109] | nurse-led eHealth cardiac rehabilitation (NeCR) | Cardiac rehabilitation (CR) is a standard treatment that can promote a healthy lifestyle, manage risk factors, improve quality of life and reduce mortality amongst people with CHD.  Information and communication technology (ICT)-based interventions are promising for the delivery of CR due to their multi-functionality, such as tele-monitoring, the uploading of health-related data for motivational feedback, video-based experiential learning and real-time social networking. | After discharge, the e-platform helped patients gain knowledge of disease management and monitor goal attainment for health behavioural changes. The nurse provided feedback on the patients’ goal attainment and lifestyle modifications on a weekly basis in a small group format through the WeChat platform, thus also mobilizing peer influence. | Nurse | Mobile app | Community/Home/Retirement home | 84 days post discharge | Once weekly | Based on the assessment data (individualized self-care needs, assess the individual's exercise capacity), the nurse and patient developed goals and action plans for lifestyle behavioural change and uploaded this information to the patient’s account on the NeCR e-platform. | The average number of website visits amongst intervention group participants who received NeCR was 8.64 (1.53) for 12 weeks intervention period. Only 31 participants uploaded data in behaviour goal-attainment, amongst which all achieved predetermined behaviour change goals except two re- ported moderate stress levels. |
| Sureshkumar (2016) [110] | Care for Stroke’ intervention | To identify operational issues encountered by study participants in using the ‘Care for Stroke’ intervention; (2) to evaluate the feasibility and acceptability of the intervention | Web-based including modules on information about stroke, home-based exercises, functional skills training, activities of daily living, and assistive devices | Not reported | Mobile app, Web-based application | Community/Home/Retirement home | 42 days post discharge | Not reported | Not reported | Not reported |
| Symer (2017) [111] | Gastrointestinal Mobile Health Application | Mobile health technology has been used in medical patients to decrease length of stay, improve glycemic control, and enhance medication compliance. | App consisted of answering survey questions (e.g., fever, vomiting, or poor oral intake) | Nurse; Primary care physician | Mobile app, Wearable device | Community/Home/Retirement home | 30 days post discharge | Daily use | Clinician responses tailored to individual if survey responses and/or patient-submitted photographs were concerning. | During the 30 postoperative days, 26 (83.9%) of patients interacted with the app by completing a survey or taking a photo at least 70% of the time, meeting our definition of feasibility. |
| Timmers (2019) [112] | The Patient Journey App | The aim of this randomized controlled trial was to investigate the effect of an interactive app on patients’ level of pain, physical functioning, quality of life, satisfaction, and health care consumption in the first four weeks of recovery after total knee replacement. | During the 28-day period after discharge, every patient in the intervention group received over 30 notifications with supporting information, related to topics such as pain, physiotherapy exercises, wound care, and daily self-care activities. Additionally, patients were requested to enter their pain scores on a weekly basis and were able to check their results within an interactive graph every week. Patients also had the opportunity to upload a photo of the wound in case of fever and an increase in pain or wound leakage. | Surgeon, researchers | Mobile app | Community/Home/Retirement home | 28 days post discharge | 1-2 notifications daily | The timing of the push notifications was configured per information item. Aligning the timing of the information to the individual patient’s phase of recovery gave it a personalized character. | In the intervention group, 93/114 (82%) patients downloaded and used the app. |
| Tolentino (2020) [113] | Meducation | Meducation® was implemented in 2017 at the participating site as a potential solution to help improve patients' medication adherence. | The app enables nurses to dynamically create fully personalized medication patient instructions in more than 20 languages, written at a 5th- to 8th-grade reading level with large font sizes, pictograms, and videos designed to make taking medications more intuitive and straightforward. | Nurse | Mobile app | Community/Home/Retirement home | Not reported | Not reported | The app enables nurses to dynamically create fully personalized medication patient instructions. | Not reported |
| Torri (2018) [114] | Cardiac Rehabilitation maintenance program (CRMP) | Although cardiac rehabilitation (CR) is cost- effective in improving the health of patients with coronary heart disease (CHD), less than half of eligible CHD patients attend a CR program. Innovative web-based technologies might improve CR delivery and utilization. | Patients were invited to record and transmit the type and amount of daily physical activity. Attending physiotherapists checked weekly compliance with medical prescriptions and workload performed, validated patient-recorded physical activity, and made customized feedback phone calls to adjust the training program, as needed. | Physiotherapy, cardiologist | Mobile app | Community/Home/Retirement home | 180 days post discharge | Not reported | Patients received a 30-day post- discharge virtual visit with a cardiologist, with measurement of body weight, waist circumference, blood pressure, and medication reconciliation and a further assessment at 90 days to present blood glucose and lipid profile results. | During the 6-mo intervention period, the intervention group had 752 web contacts (average 30) through the dedicated chat and 70% had at least 1 video conference with health care professionals at the cardiac rehabilitation unit. Active daily data transmission was 100% during the first month, 88% at 3 mo, and 81% at 6 mo. Most patients (85%) completed at least 80% of the prescribed intervention (i.e., at least 4.8 months of interactive contacts with health care professionals). |
| van den Berg (2016) [115] | CARE4STROKE | We hypothesized that an 8-week program of CME, commenced in hospital, supported by e-health and combined with tele-rehabilitation after discharge, results in improved self- reported mobility and reduced length of stay (LOS) without increased levels of strain of the caregiver when compared with usual care. | In brief, the patient and carer were provided with an iPad which was loaded with the caregiver-mediated exercises (CME) application with 37 standardized exercises aimed to improve gait and gait-related mobility, such as standing, turning, or making transfers. Additionally, participants in the intervention group wore an activity monitor the Fitbit Zip that monitors physical activity, and it was used to motivate participants to increase physical activity through real-time feedback. | Physiotherapy | Mobile app, Wearable device | Community/Home/Retirement home | 56 days post discharge | 5 times a week for 30 minutes | The intervention comprised an 8-week caregiver-mediated training program with support using a customized exercise app loaded onto a tablet. | Twenty out of 31 patients in the intervention group received the tele-rehabilitation at home. Seven patients remained in hospital for the full length of the intervention period, and four patients withdrew from the study. |
| Venkatraman (2022) [116] | ManageMySurgery (MMS) | Transcatheter aortic valve replacement (TAVR) is a less invasive alternative to open surgery that is becoming more prevalent. However, no peer‐reviewed research has established the feasibility of a comprehensive, longitudinal software platform for pre‐, peri‐, and post‐TAVR management. | ManageMySurgery (MMS) combines patient education and outcomes tracking for patients undergoing transcatheter aortic valve replacement (TAVR) procedures. Pre‐ and postoperatively, patients completed app‐based tasks and reported clinical results using validated New York Heart Association (NYHA) and the Kansas City Cardiomyopathy Questionnaire (KCCQ‐12) surveys. | clinical staff member/ research team | Mobile app | Community/Home/Retirement home | 30 days post discharge | Survey administered pre-operatively and 1 month post-operatively | Not reported | Users logged on to the application with their username and password an average of 2.6 times, with 34 (79.1%) users signing in one to three times. Because users could securely access content repeatedly without signing in to the application by signing in to their phone, we were not able to capture the total number of times the patients opened the application |
| Vincent (2021) [117] | My Hip Fracture (My-HF) | Mortality and morbidity are high for older adults after hip fracture (HF), but patients and surrogate decision makers (SDMs) are typically unaware of the poor prognosis. We developed a novel educational tool, My Hip Fracture (My-HF), to provide patients and SDMs of patients hospitalized with acute HF individualized estimates of their post-HF prognosis. | We designed My-HF to facilitate communication of individualized risk of 2 key adverse outcomes in a user-friendly, patient-centered fashion: 1) death or major complication within 30 days of surgery and 2) discharge to post-acute care (rehabilitation or long-term care). My-HF provides information about: 1) anatomy and risk factors for HF; 2) Hip fracture treatment received; 3) individualized predicted risk of adverse events and 4) anticipated discharge trajectory. | Not reported | Mobile app | Community/Home/Retirement home | Not reported | Not reported | My Hip Fracture (My-HF), provides patients and surrogate decision makers (SDMs) of patients hospitalized with acute Hip Fracture (HF) individualized estimates of their post-HF prognosis. | Not reported |
| Visperas (2021) [118] | JointCOACH | Technologies, including web-based platforms used to enhance patient education and communication, are being explored to improve patient engagement, satisfaction, and potentially reduce cost. These platforms are designed to educate and follow patients more closely by assessing for “red flags” and alerting the care team when increased monitoring is suggested. | This study tested a web-based interactive patient-provider software platform (IPSP), which enabled patient communication with their care team and preparatory/recovery guidance. Through this IPSP, patients receive information at key intervals focusing on the following: (1) instructions about how to prepare for surgery, (2) information about the surgery, (3) information about medications and pain control, and (4) information about postoperative recovery and rehabilitation. | Nurse, Primary care physician, Surgeon | Web-based application | Community/Home/Retirement home | 90 days post discharge | Not reported | Care team followed up with patients within 24 hours of being alerted by the IPSP (interactive patient-provider software platform) through telephone call or written response. | Overall, for patients on the intervention arm of the study, 87% of patients activated the interactive patient-provider software platform (IPSP). Of those, there was a 90% compliance rate (calculated as the total number of forms completed out of total number of forms sent to patients during the combined preoperative and postoperative period) and 97% of patients felt they were pre- pared for surgery. |
| Vloothuis (2019) [119] | CARE4STROKE digital intervention | To improve functional outcome and to facilitate early supported discharge by increasing the intensity of task specific training using an 8-week caregiver-mediated exercise program with e-health support after stroke | Control group: Usual stroke care Intervention group: Trained an additional amount of 150 minutes a week caregiver-mediated exercises program with e-health support, combined with telerehabilitation, in addition to usual care.  Exercises chosen from 37 standardized exercises aimed at improving mobility. | Physiotherapy | Telephone, Video conferencing or Email | Community/Home/Retirement home | 56 days post discharge | Perform the selected set of exercises at least five times a week for 30 minutes | For each patient, exercises were combined into a patient-tailored, progressive training regimen, related to the patient goals | Not reported |
| Vonk Noordegraaf (2014) [120] | Personalised eHealth programme after gynaecological surgery | To evaluate the effectiveness of an eHealth intervention on recovery and return to work, after gynaecological surgery | Achieving self-empowerment; Employer and care provider communication; Identify recovery problems; Surgical procedure information; Extensive list of frequently asked questions; A forum for women to connect | Nurse, Gynaecologists | Web-based application | Community/Home/Retirement home | Not reported | 49 | Tailored instructions on resuming daily activities, including work, and tools to improve self-empowerment and identify recovery problems before and after surgery were provided | Adherence not reported. Number of women who completed secondary outcomes measures at 2, 6, 12, and 26 weeks was 97, 97, 96, and 97%, respectively. |
| Wang (2017) [121] | Web-based coaching program using EHRs | To assess the effectiveness of continuous post discharge care in helping patients to maintain good health. | COPD lifestyle education;  Medication guidance; Education;  Pulmonary rehabilitation instructions; Participant access to EHRs; Ability to read all collected information and discuss with administrators via internet; Assess participant management based on progress of disease. | Nurse, Primary care physician | Web-based application | Community/Home/Retirement home | 365 days post discharge | Frequently with prompts every two weeks | Tailored contents to match a user’s preference. Components of tailoring not reported | Follow-up visits at 1, 3, 6, and 12 months to promote adherence. n=10 lost at 12months follow-up |
| Wang (2018) [122] | Mobile app stoma home care | To explore the effects of a home care mobile app on the outcomes of stoma patients who discharged from hospital | control group (n = 103) : Routine care such as written and verbal health education on preoperative preparation, stoma surgery and postoperative stoma care. Intervention group (n = 100): Routine care and Home care via a mobile app such as receive stoma care from ET nurses through this mobile app at home. App components included  - appointment  - photograph diagnosis  - consultation | Nurse | Mobile app | Community/Home/Retirement home | 183 days post discharge | Not reported | Not reported | Adherence not reported. 9 participants reportedly lost to follow-up |
| Werhahn (2019) [123] | Cardio Patient Monitoring Platform (CPMP) | Tele-monitoring concepts have been shown to reduce hospital readmissions/admissions and to improve further heart failure outcomes like all-cause mortality and health-related quality of life (QoL). Mobile health (mHealth) applications proved useful to provide assistance in self-management, symptom monitoring, and home-based cardiac rehabilitation. Thus, we developed the cardio patient monitoring platform (CPMP) for smart device-based monitoring of HF patients and examined its performance towards usability as mHealth concept. | It allowed for safe and continuous data transmission of self-measured physiological parameters (blood pressure, body weight), activity data, and patient-reported symptoms. Additional app features including reminders/confirmation of medication intake or input of symptoms were offered to examine acceptance and functionality but were not mandatory. | Primary care physician | Mobile app, Wearable device | Community/Home/Retirement home | 60 days post discharge | Once daily | Data were checked daily, and a response was initiated within 24 h when predefined margins in blood pressure were exceeded, increase in body weight, or new or worsening signs or symptoms occurred. | Patient adherence with daily intake of self-measured data was satisfactory (82.95% for blood pressure and 78.18% for body weight). |
| Willeit (2020) [124] | MyStrokecard | It aimed to (i) reduce one-year risk of major cardiovascular events and (ii) improve health-related quality of life one year after the index event. | "MyStrokeCard" provided risk factor monitoring, ascertainment of post-stroke complications, and extended patient education and were offered to contact the study personnel in case of health problems. | Physiotherapy, Occupational therapist, Speech therapist, Nurse, Primary care physician | Web-based application | Community/Home/Retirement home | 365 days post discharge | Not reported | They received training for this e- tool during hospital stay with a tailored composition according to individual risk profiles and target levels and introduction to easily applicable screening tools for post-stroke complications. | When observing a lower-than-expected attrition rate (including withdrawals of consent and losses to follow-up) between January 2014 and June 2017, the target sample size was revised to 2160 patients. Initially planned to enrol 2400 patients. |
