# Supplementary material for: Platform-Based Patient-Clinician Digital Health Interventions for Care Transitions: Scoping Review
Source: J Med Internet Res. 2024 Dec 30;26:e55753. doi: 10.2196/55753 (PMC11729789; doi:10.2196/55753)
Supplement: Multimedia Appendix 5 [file jmir_v26i1e55753_app5.docx]

**Supplementary Table 4. Barriers and enablers for the uptake and implementation of DHIs**

| **Barriers (n=8)** | **Patients /caregivers** | **Providers** |
| --- | --- | --- |
| Lack of interest   - Low motivation, living alone or without long-term nursing care. | [28,34,56,63,64,70,76,89,95,106,107,118,124] | **---** |
| Time constraints   - Difficulty focusing on/looking at device, too tired. - Perceived to be an external burden. | [54,56,78,79,96,106,109,115] | [44,56,76] |
| Technological issues   - Screen size too small, incompatible operating system, no record of username / password. - Patients unable to achieve adequate connectivity at home; others thought the DHIs were too demanding and required too much information. - DHIs registration difficulties. - Wireless connectivity problems, difficulty uploading photos at the appropriate time. - Difficulty contacting patients, programming errors, in-app advertisements. - Server-sided problems with clinicians viewing patient data. - Timing, lack of smartphone password leading to delay in app download. | [41,54,62,79,88,90,111] | [90,96,111] |
| Usability issues   - Remembering to undertake the task. | [42,62,71,96,107,117] | [76] |
| Language barrier | [28,66,89] | [96] |
| Content of DHIs not relevant   - Disliked app content. - Patients unaware of the source of the content, not adaptive to patient. attitudes, concerns, or perceptions. - Concern with usefulness. | [96,106,117] |  |
| Lack of comfort   - Feeling overwhelmed, unable to find a carer to assist them. - Difficulty with medical jargon, unfamiliar terms (e.g., comfort care), and with certain graphics). | [78,115,117] | --- |
| Lack of support and engagement | **----** | [106] |
| **Enablers (n=7)** |  |  |
| Able to use / Able to use own device / Able to use App | [28,36,62,64,67,70,76,78,80,81,89,96,99,101,111,116,117] | --- |
| Easy to use   - Low stress levels reported. - Own a smartphone. | [49,55,57,58,68,76,85,101,105,111,121] | [57] |
| Ability to collaborate with patients | **---** | [44] |
| Caregiver support | [115] | --- |
| Confidence in technology   - Believe that website helped in evaluating symptoms and deciding if health professionals are needed, believe website helped reduce worry about the recovery process). | [51] | --- |
| Convenience of using the DHIs   - DHIs were more convenient than phone calls. | **---** | [62] |
| Participating in the development and implementation processes | **---** | [45] |
| **Both barrier and enabler (n=3)** |  |  |
| Limited or no access to technology/internet (Barrier) | [28,36,51,54,56,61,63,70,75,90,99,10,107,111,118] | --- |
| Access to technology / internet (Enabler) | [28,39,42,51,54,58,61,64,65,67,70,75,76,79,80,81,82,90,93,95,99,101,105,109,112,116,118,121] | --- |
| Difficulty with understanding the DHIs (Barrier)   - Felt overloaded by the context of their disease or the complexity of the system. | [28,49,64,117] | **---** |
| Easy to understand (Enabler)   - Helped prepare for discharge, application well organized. - Interested in the DHIs - The novelty of new technology | [31,33,34,38,49,55,57,58,68,76,80,81,85,87,93,101,105,111,121] | [57,76] |
| Lack of digital literacy (Barrier) | [31,36,59,63,64,89,99,101,107,112,118] | **---** |
| Digital literacy (Enabler) | [39,40,42,58,59,64,65,76,78,82,90,99,101,109,116,118,123] | --- |
